# Hypertensive disorders of pregnancy in low- and middle-income countries: a review of antenatal interventions to improve maternal outcomes

**DOI:** 10.3389/fgwh.2026.1848249

**Published:** 2026-06-24

**Authors:** Kristen Riley, Lily Yan, Marie Marcelle Deschamps, Anju Ogyu, Wheytnie Alexandre, Line Malha, Rachel Sinkey, Jean William Pape, Vanessa Rouzier, Margaret McNairy

**Affiliations:** 1 Weill Cornell Medicine, New York, NY, United States; 2 GHESKIO Centers, Port-au-Prince, Haiti; 3 Washington University in St. Louis, St. Louis, MO, United States; 4 The University of Alabama at Birmingham, Birmingham, AL, United States

**Keywords:** hypertensive disorders of pregnancy (HDP), interventions to improve maternal outcomes, low- and middle-income countries, maternal health, maternal mortality and morbidity

## Abstract

**Introduction:**

Hypertensive disorders of pregnancy remain a major contributor to maternal mortality worldwide, responsible for an estimated 16% of maternal deaths with the majority in low- and middle-income countries (LMICs). This review synthesizes interventions for HDP tailored to LMICs to improve their widespread implementation and in turn, improve maternal outcomes.

**Methods:**

This review synthesizes literature published between 2010 and 2024 on interventions addressing hypertensive disorders of pregnancy in LMICs using MEDLINE, Cochrane, EMBASE, and WHO Global Index Medicus. Interventions are described using a novel conceptual framework establishing three key steps of the hypertension disorders of pregnancy care continuum: prevention, screening, and treatment.

**Results:**

A total of 212 studies were identified with interventions across multiple domains: 21 involved (10%) screening, 51 (24%) prevention and 119 (56%) treatment, and 21 (10%) including more than one step. The majority of studies were from lower-middle- and middle-income countries, with less than 10% including only low-income countries. Interventions range from educational programs, clinical trials evaluating medication type and dosing algorithms, and development of novel screening tools with comparably little implementation science studies.

**Discussion:**

The majority of studies on interventions for hypertensive disorders of pregnancy in LMICs were randomized trials focused on efficacy. While there were several feasibility and acceptability studies, there was a lack of research translating trials to real-world community and clinical settings. Further, interventions were lacking in countries with the highest rates of maternal mortality. Additional research should focus on the implementation of integrated strategies that improve care across all stages of the care continuum, particularly in LMICs with the greatest burden.

**Systematic Review Registration:**

https://osf.io/7sz5w/overview.

## 1 Introduction

Low- and middle-income countries (LMICs) bear 95% of global maternal deaths (1). In 2020, the global maternal mortality ratio was 223 deaths/100,000 live births—nearly 3-fold higher than the United Nations Sustainability Goal of less than 70 maternal deaths per 100,000 live births by 2030 (2). Hypertensive disorders of pregnancy (HDP) are a leading cause of preventable maternal morbidity and mortality and include gestational hypertension, preeclampsia, eclampsia, and chronic hypertension (3, 4). HDP increase the risk of maternal morbidity such as stroke and placental abruption and bear long-term consequences including chronic hypertension, increased long term cardiovascular, and stroke risk. HDP are also associated with fetal and neonatal morbidity such as prematurity, need for neonatal intensive care and low birth weight (5). Rates of HDP in LMICs are disproportionately high and increasing (6). In Latin America and the Caribbean, HDP are estimated to cause 26% of maternal deaths, as compared to 16% in developed countries (7). Reasons for high rates of HDP-related maternal death are hypothesized to be gaps in screening, diagnosis, and treatment (8). This review addresses these critical gaps by examining strategies to improve timely identification and management of HDP in resource-limited settings to improve maternal outcomes.

This review synthesizes the literature on interventions to improve the prevention, screening, and management of HDP that have been developed and implemented in LMICs. It identifies current gaps in the literature and opportunities for further investigation. A global understanding of interventions for HDP to improve care for pregnant individuals will allow these interventions to be more easily scaled and adapted across LMICs, with the ultimate goal of improving direct and long-term maternal health.

## 2 Methods

### 2.1 Search strategy and selection criteria

Using the methodological framework proposed by Arksey and O’Malley, a review was performed on interventions to care for HDP in LMICs (9). Original peer-reviewed articles published in English from January 2010 to April 2024 were collated from systematic search of four databases, including: MEDLINE, Cochrane, EMBASE, and WHO Global Index Medicus. The final search strategies can be found in Supplementary File 1. All identified articles were compiled in review software, Covidence.

The search strategy was developed using a number of core concepts and associated keywords. The first core concept aimed at identifying the appropriate conditions, broadly, hypertensive disorders of pregnancy, for which search terms such as “hypertension” and “eclampsia” were used. The second concept aimed at limiting the search to pregnant patients, using terms such as “antepartum” and “pregnancy”. The third concept focused on identifying interventions, including both known evidence-based strategies, such as “aspirin” and “magnesium” but also broader terms such as “management” and “treatment”. The final key concept aimed to limit studies to LMICs using terms such as “LMIC” and “developing nation” but also using specific country names (as outlined below). See Supplementary File 1 for an example search strategy.

To be included, articles had to meet the following criteria. First, only peer-reviewed articles were included. Unpublished or gray literature as well as review articles or studies outlining protocols were excluded. Second, only articles published in English were included. Third, studies published between 2010 and 2024 were considered, given that a literature review on the diagnosis, management, and prevention of HDP published in 2010 included studies through 2009 (8). Fourth, only studies with a primary study population in an LMICs were included. LMICs were defined by the World Bank Country and Lending Groups country classification; countries categorized as “low-income economies”, “lower-middle-income economies”, and “upper-middle-income economies” were included. To account for changing classifications over time, the Cochrane Effective Practice and Organisation of Care (EPOC) LMIC filter was used. Finally, all included studies focused on interventions to improve care for HDP defined as gestational hypertension, chronic hypertension, preeclampsia, eclampsia, and chronic hypertension with superimposed preeclampsia or eclampsia were considered. Additionally, studies focused on preventing HDP in pregnant women not diagnosed with HDP were also included. As this review focused primarily on hypertension during pregnancy, studies addressing the management of new-onset hypertension during the postpartum period were excluded.

### 2.2 Study selection and data extraction

**Figure 1:**
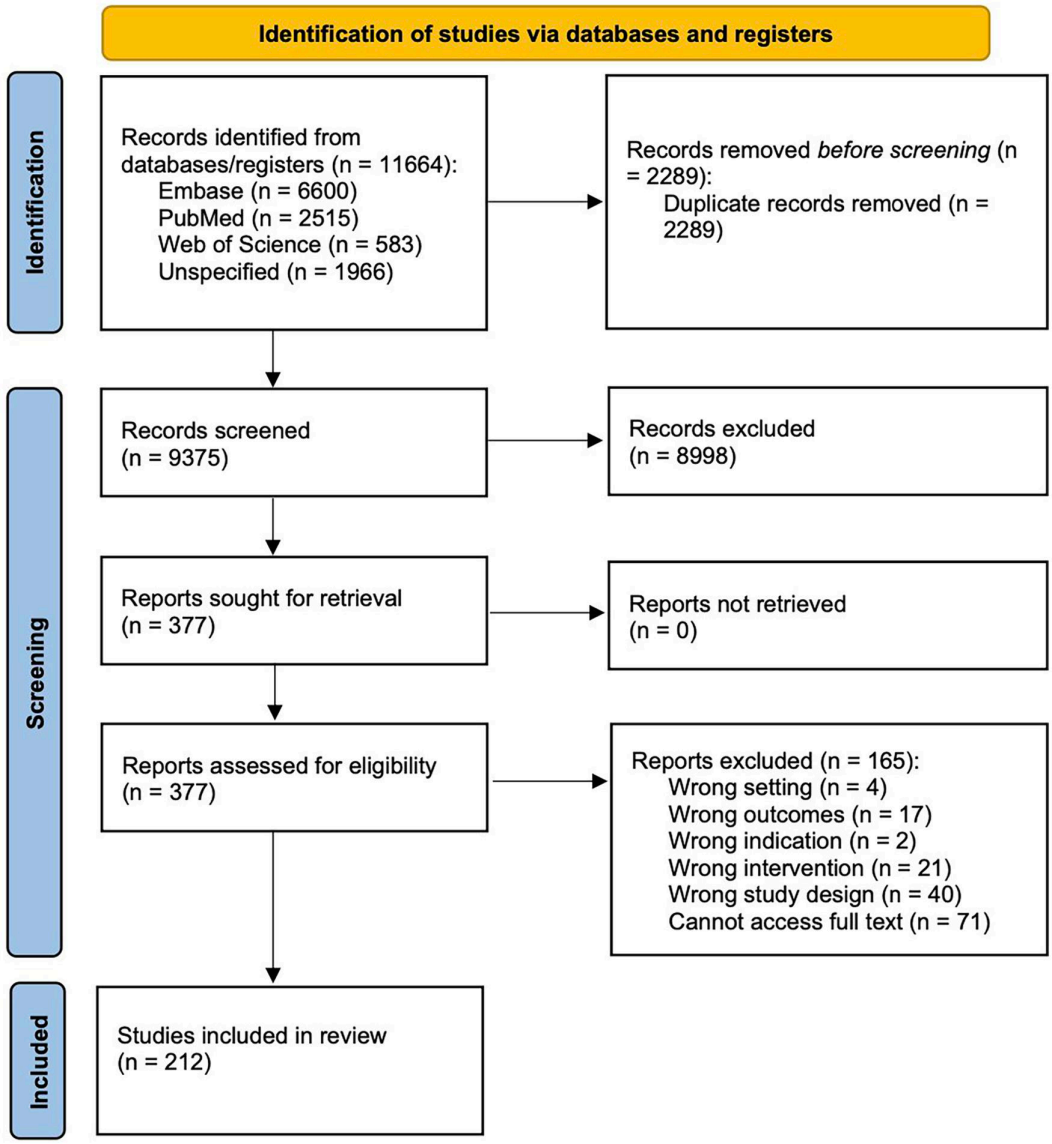
Flow chart of study identification and inclusion process. *Wrong setting: studies not conducted in LMICs. Wrong outcomes: studies that did not assess maternal outcomes. Wrong indication: studies including conditions other than HDP. Wrong intervention: studies that did not describe a specific intervention. Wrong study design: comment papers, review articles, and study protocols.

Following compilation of identified articles, one reviewer screened the articles using the outlined eligibility criteria. First, articles were screened using titles and abstracts followed by full text screening (Figure 1). Once articles fulfilling the eligibility criteria had been identified, each article was reviewed for data extraction. Study details, including title, year of publication, author(s), country, and journal of publication were extracted. In addition, methodological details of each study were recorded, such as study aims, study design, study duration, number of participants, type of hypertensive disorders of pregnancy studied, diagnostic criteria used for HDP, stage of the care continuum addressed, inclusion criteria, exclusion criteria, and demographic details. Finally, information regarding the intervention and outcomes were recorded, including details of the intervention, duration and frequency of the intervention, the control or comparison group, primary and secondary outcome(s), important results, and conclusions or implications of the study results.

### 2.3 Synthesizing and summarizing the data

Quantitative and qualitative data were synthesized from studies. Quantitative data included the number, geographical distribution, study design, and year of publication of included studies. Studies were grouped based on the stage of the care continuum that they addressed, including prevention, screening, and treatment; within each stage, themes were identified using a grounded theory approach.

## 3 Results

**Figure 2:**
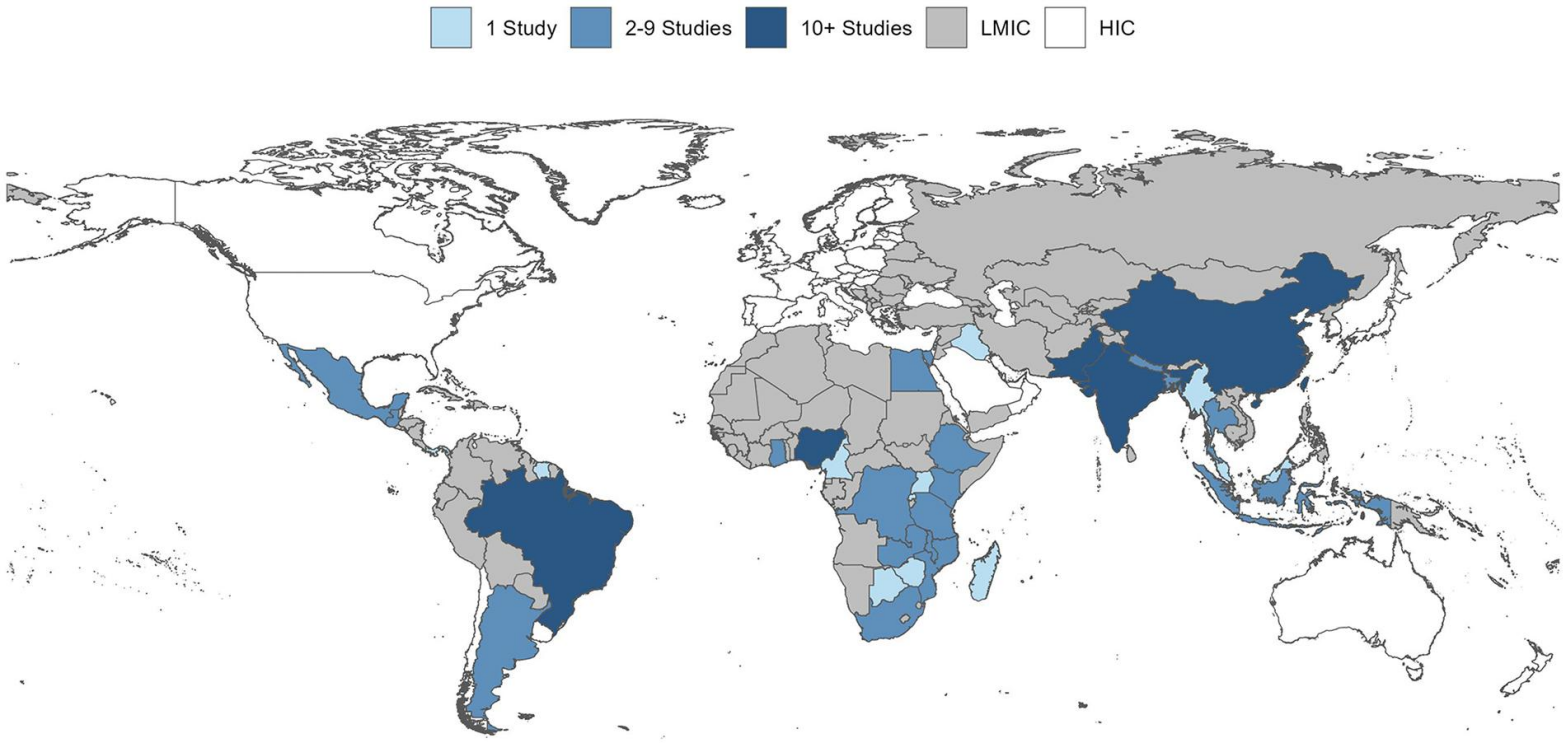
Number of studies by country.

This review identified 212 studies focused on interventions for HDP among women in LMICs (Table 1). A total of 51 studies focused on prevention of HDP (24%), 21 on screening (10%), 119 on treatment (56%), and 21 on multiple stages (10%). From 2010 to 2024, the number of studies increased steadily with a 250% increase from 2010 to 2023. Overall, approximately half of studies were clinical trials and about one-fourth were cohort studies. The majority were concentrated in the countries of China, India, and Nigeria (Figure 2).

**Table 1:** Sample of included studies by stage of the care Continuum.

Author Year Country	Design	Intervention; Control	Maternal Outcomes
Prevention (*N* = 57)			
Calcium Supplementation			
Thapa et al. (16) 2016 Nepal	Prospective Cohort Study	Intervention: District-level orientation for ANC providers on antenatal calcium intervention, consisting of education and counseling for pregnant women, provision of Ca, consumption of 1 g Ca daily from 4 months gestation for 5 months or until delivery Control: NA	100% of women were counseled on Ca use and received Ca. 99.2% of women reported taking the correct dose with correct timing. 91.4% of women consumed the entire course of Ca.
Dwarkanath et al. (21) 2024 India and Tanzania	Randomized Controlled Trial	Intervention: 500-mg Ca supplementation (1 Ca tablet, 2 placebo tablets) Control:1,500-mg Ca supplementation (3 Ca tablets)	In both India and Tanzania, 500-mg Ca was noninferior to 1,500-mg Ca in incidence of pre-eclampsia. In India, but not Tanzania, 500-mg Ca was noninferior to 1,500-mg Ca in incidence of preterm birth.
Omotayo et al. (17) 2018 Kenya	Mixed-Methods Study	Intervention: Training for healthcare workers on Ca supplementation Control: NA	80% of ANC clients reported being counseled about Ca and IFA supplementation. 83% adherence was recorded for Ca supplementation.
Low-Dose Aspirin			
Kumar et al. (27) 2020 India	Randomized Controlled Trial	Intervention: 150 mg aspirin daily in women at high risk of pre-eclampsia Control: 75 mg of aspirin daily in women at high risk of pre-eclampsia	Pre-eclampsia occurred in 6.5% of women of the 150 mg aspirin group, which was significantly lower than the 17% of women in the 75 mg aspirin group.
Hoffman et al. (77) 2020 India, Democratic Republic of the Congo, Guatemala, Kenya, Pakistan, Zambia	Randomized Controlled Trial	Intervention: 81 mg of aspirin daily in nulliparous pregnant women 18–40 compared Control: placebo daily	There were no differences in overall rate of hypertensive disorders of pregnancy between groups.
Vitamins/Supplements			
Kabuyanga et al. (31) 2024 Democratic Republic of Congo	Randomized Controlled Trial	Intervention: Vitamin D supplementation with a single monthly oral dose of 60,000 IU for six months initiated prior to 16 weeks gestation Control: NA	The incidence of pre-eclampsia was in the vitamin D group was 2.1%, significantly lower than 5.7% in the non-supplemented group. Vitamin D supplementation significantly reduced the risk of caesarean delivery by 37%. Risk of induction of labor was forty-times higher in the non-supplemented group than the supplemented group.
Liu et al. (29) 2021 China	Randomized controlled trial	Intervention: Comparing pregnancy-induced hypertension in women receiving supplementation with multiple micronutrients (UNIMMAP supplement), iron and folic acid, or folic acid alone at <12 weeks versus at ≥12 weeks gestation Control: NA	When comparing women who took iron-folic acid or folic acid alone at <12 weeks versus ≥12 weeks, there was no significant difference in risk of pregnancy-induced hypertension. However, early consumption of multiple micronutrients showed a 26% risk reduction for pregnancy-induced hypertension.
Screening (*N* = 41)			
Community Engagement			
Sevene et al. (35) 2020 Mozambique	Cluster randomized controlled trial	Intervention: Community-level interventions for pre-eclampsia (CLIP) intervention package, consisting of: Community health worker-led visits with women using the PIERS On the Move (POM) mobile health application with a risk stratification tool Community engagement sessions with community leaders, women, and family members Control: routine antenatal care provided by nurses, doctors, and community health workers	Women in the intervention group received significantly more routine antenatal care compared with the control group. However, <50% of women received ≥4 routine antenatal care visits in both groups. Between groups, compliance with BP, proteinuria measurement, and the composite primary outcome (all-cause maternal, fetal, and newborn mortality and major morbidity) was not significantly different.
Mahmoud et al. (78) 2023 Nigeria	Qualitative study	Intervention: Describe the barriers and facilitators to current and optimal management of hypertensive disorders of pregnancy, focusing on implementation of home blood pressure monitoring, through interviews with healthcare workers and patients Control: NA	Facilitators included perceived simplicity, high burden of HDP, availability of a multi-disciplinary team, protocols used for other conditions, and availability of mobile technology. Barriers included limited knowledge of HDP among patients and providers, socioeconomic challenges, limited transportation, inadequate equipment, and prevailing religious and sociocultural norms. 81–88% of participants reported that a blood pressure monitoring program would be acceptable. 56–72% reported that it would be appropriate. 47–69% reported that it would be feasible.
Healthcare Provider Training			
Toledo-Jaldin et al. (39) 2023 Bolivia	Case-control study	Intervention: HDP diagnosis before and after a workshop with healthcare providers reviewing the ACOG guidelines for diagnosis of HDP Control: NA	Utilization of ACOG guidelines increased after the workshop. After the workshop, there were fewer gestational hypertension and eclampsia cases, but more cases of pre-eclampsia and severe pre-eclampsia. After the workshop, more pre-eclampsia cases were identified as having proteinuria and more severe pre-eclampsia cases were identified with abnormal laboratory values rather than clinical symptoms.
Maaløe et al. (41) 2019 Zanzibar	Retrospective study	Intervention: pre- and post-implementation of the PartoMa intervention and pocket guide. PartoMa seminars were conducted with doctors and nurse midwives to establish use of guidelines via case-based discussions. One page of the pocket guide and one of five seminar cases focused on intrapartum care for women with severe HDP. Control: NA	The routine BP assessments of all laboring women did not improve between the two periods, however, diagnosis of severe HDP and proteinuria testing improved. The proportion of women with at least one recorded BP post-delivery improved from 67.0% at baseline to 77.0% during the intervention months. Among the women with severe hypertension, initiation of antihypertensive treatment increased, from 46.7% women at baseline to 64.2% during the intervention months. In both periods, 90% of women with severe pre-eclampsia/eclampsia had magnesium sulfate treatment initiated.
Quality Assessment			
Rawlins et al. (42) 2018 Ethiopia, Kenya, Madagascar, Mozambique, Rwanda, Tanzania	Cross-sectional study	Intervention: Quality of care health facility assessment to measure adherence to WHO standards for screening and management of pre-eclampsia and eclampsia Control: NA	BP assessment was relatively high with 68% of ANC clients having BP taken with proper technique (ranging from 46% in Rwanda to 96% in Kenya). Less than a quarter (24%) of women admitted to labor and delivery services were asked about signs of preeclampsia/eclampsia (ranging from 11% in Mozambique to 34% Kenya and Ethiopia). Availability of MgSO4 in health centers varied, ranging from 4% in Rwanda to 96% in Mozambique.
Mobile Health			
Parsa et al. (43) 2019 Iran	Prospective cohort study	Intervention: Mobile application consisting of educational topics related to pre-eclampsia, including definition, risk factors, complications, signs and symptoms. A questionnaire was administered to pregnant women before and after implementation in the intervention group, who used the application Control: Questionnaire administered to control group of pregnant women who did not use the application	Mean knowledge scores were not significantly different between the intervention and control groups before the intervention. However, after the intervention, mean knowledge scores in the intervention group were significantly higher than in the control group (78.08 +/- 14.19 vs. 15.75 +/- 19.49; *p* < 0.001).
Treatment (*N* = 139)			
Magnesium			
Budhwani et al. (47) 2017 India	Retrospective cohort study	Intervention: Use of magnesium sulfate amongst emergency obstetric care trainees Control: NA	Of the training centers with at least one reported pre-eclampsia/eclampsia case, 57% reported at least one use of magnesium. The proportion of patients with eclampsia that received magnesium in each state varied from 12 to 76% with lower rates in rural communities.
Beyuo et al. (55) 2022 Ghana	Randomized Controlled Trial	Intervention:12-hour intervention group of MgSO4 (loading dose of 4 g IV MgSO4 and 10 g IM MgSO4 followed by 5 g IM MgSO4 every 4 h for a total of 3 maintenance doses) Control: 24-hour control group (loading dose of 4 g IV MgSO4 and 10 g IM MgSO4 followed by 5 g IM MgSO4 every 4 h for a total of 6 maintenance doses)	There was no statistically significant difference in occurrence of seizures, maximum blood pressure, or adverse maternal outcomes between the two groups. Length of inpatient admission, duration of urethral catheterization, and rates of side effects were lower in the 12-hour group.
Okonofua et al. (52) 2013 Nigeria	Multi-Center Intervention	Intervention: Training for doctors and midwives on using magnesium sulfate for the management of eclampsia Workshops included review of epidemiology, clinical features of eclampsia, ways to overcome health system limitations, quality of care, and protocols for treatment with magnesium sulfate Control: NA	The overall case fatality rate declined from 15.1% before the intervention to 3.2% after the intervention (*p* < 0.001). The overall maternal mortality rate declined after the intervention, but this difference was not statistically significant.
Antihypertensive Medications			
Salama et al. (45) 2019 Egypt	Randomized Controlled Trial	Intervention: methyldopa (1–2 g per day) versus nifedipine (20–40 mg per day) in patients with mild to moderate chronic hypertension Control: no antihypertensive (placebo, vitamin C tablets) in patients with mild to moderate chronic hypertension	Mothers in the no medication group were more prone to develop severe hypertension, pre-eclampsia, renal impairment, electrocardiogram changes, placental abruption, and repeated hospital admissions (*p* < 0.001) compared to both medication groups.
Raheem et al. (44) 2012 Malaysia	Randomized Controlled Trial	Intervention: time and doses to target BP in women with sustained severe hypertension (SBP≥160 or DBP≥110 measured on at least two occasions in 4 h) treated with IV labetalol Control: time and doses to target BP in women with sustained severe hypertension (SBP≥160 or DBP≥110 measured on at least two occasions in 4 h) treated with oral nifedipine	The median time to achieve target BP was shorter in the nifedipine group (30 min) than the labetalol group (45 min) but this difference was not statistically significant. There were no significant differences in number of doses required to achieve target BP or pregnancy outcomes.
Adebayo et al. (79) 2020 Nigeria	Randomized Controlled Trial	Intervention: oral nifedipine (20 mg every 30 min until target BP or maximum of 5 doses) in patients with severe hypertension Control: IV hydralazine (10 mg every 30 min or until target BP or maximum of 5 doses) in patients with severe hypertension	Acute control of BP was faster in the hydralazine group (44 min) compared with the nifedipine group (51 min), but this difference was not significant. The average doses required to control BP was significantly lower in the nifedipine group. Risk of recurrence and retreatment of severe hypertension was significantly lower in the nifedipine group.
Expectant Management			
Swamy et al. (66) 2012 India	Prospective cohort study	Intervention: Examined days of pregnancy prolongation in women with severe pre-eclampsia who were managed with expectant management and non-invasive monitoring Control: NA	The days of pregnancy gained were significantly higher among those who had expectant management between 28.1 and 30 weeks (14 days) compared with 30.1–32 weeks (5 days) and 32.1–34 weeks (4 days). There were no instances of maternal death, cerebrovascular accident, or severe acute renal failure among the women managed expectantly. Increasing gestational age correlated with reduction of respiratory distress syndrome.
Alternative Therapies			
Sahoo et al. (69) 2022 India	Randomized Controlled Trial	Intervention: Benson's relaxation therapy, where the woman breathed quietly with eyes closed for 15–20 min twice daily, in conjunction with antihypertensives in women with pregnancy-induced hypertension Control: antihypertensives alone in women with pregnancy-induced hypertension	The relaxation group demonstrated a larger decrease in systolic blood pressure (mean difference: control 5.9 +/- 5.5, intervention 14.6 +/- 3.8, *p* = 0.000) and diastolic (mean difference: control 5.8 +/- 7.3, intervention 14.0 +/- 3.7, *p* = 0.000) blood pressure following the intervention compared with the control group.

HDP, hypertensive disorders of pregnancy; ACOG, American College of Obstetricians and Gynecologists; BP, blood pressure; WHO, World Health Organization; ANC, antenatal care; MgSO4, magnesium sulfate; SBP, systolic blood pressure; DBP, diastolic blood pressure; IV, intravenous.

### 3.1 Prevention

Of the 57 studies addressing interventions to improve prevention of HDP, the majority (46 studies) focused on pharmacologic interventions, with 19 on calcium supplementation, 17 on low-dose aspirin administration, and 10 on vitamins and supplements (Table 1).

#### 3.1.1 Calcium

Calcium supplementation for prevention of preeclampsia is recommended by WHO at a dose of 1.5–2.0 grams daily in areas with low dietary calcium intake (10). In the included studies, rates of calcium supplementation in pregnant women in select LMICs with low dietary calcium uptake range from 0%–73%. (11–13) Reasons for not taking calcium include side effects and palatability, stigma of taking pills, and lack of patient and provider knowledge as well as overburdened staff (12, 14, 15). Facilitators to calcium supplementation adherence include reminders from family members and improving supplement taste (15). Few studies described targeted interventions to improve calcium supplement intake. One qualitative study in Kenya utilized adherence partners to remind women to take supplements; women report that these partners positively impacted adherence (15). Two studies implemented trainings for antenatal care providers to educate patients on calcium supplementation; both interventions were effective in achieving high coverage and compliance (16, 17). Two studies evaluated the type of calcium supplement used, aiming to optimize palatability. Studies are not unanimous about preferred type of tablet to maximize adherence. One study in Bangladesh found that the majority of women preferred conventional tablets (62%) followed by chewable tablets (19%) (18). A second study, conducted in Kenya, demonstrated preference for chewable tablets over hard tablets (19). One study in Bangladesh assessed the impact of low-dose (500 mg) calcium supplementation on blood pressure as a part of a community health worker-led maternal nutrition initiative; it found that women who had 4 or more antenatal care visits were more likely to take calcium supplements. It also demonstrated a 46% reduction in hypertension in women who consumed the calcium for more than 6 months of pregnancy compared to those who consumed the calcium for fewer than 3 months (20). However, this improvement in outcomes may have been driven by improved antenatal care in those taking supplements for longer. Finally, a number of studies examined the optimal dose for calcium supplementation to prevent preeclampsia. A study from India and Tanzania compared a 500-mg dose daily to the traditional 1500-mg dose daily. In both settings, the majority of participants had baseline dietary calcium intake <800 mg. This study demonstrated noninferiority of the lower dose in reducing risk of preeclampsia in both countries but only showed noninferiority of the lower dose in reducing risk of pre-term birth in India (21).

#### 3.1.2 Low-dose aspirin

Low-dose aspirin (LDA) has been shown to reduce rates of preeclampsia, premature delivery, fetal growth restriction, and postpartum hemorrhage in women with risk factors for preeclampsia and eclampsia (22). The prophylactic benefit of LDA is more pronounced in populations at high risk for preeclampsia or adverse pregnancy outcomes. This high-risk population may be defined differently across different national and international organizations. One study in China among 291 women reported 86% of women having compliance <50% with LDA (23). Studies found rates of LDA prescription to range widely which may in part account for disparities regarding LDA candidacy recommendation across guidelines. In Thailand, LDA prescription ranged from 50%–90% depending on the risk factor for preeclampsia present (24). In a study in Ethiopia amongst women diagnosed with preeclampsia, only 1.1% of women with an indication for aspirin prophylaxis had received aspirin prior to development of preeclampsia. This study notably required that women have one risk factor for preeclampsia for LDA to be indicated, compared to WHO guidelines which require two or more risk factors (22, 25). In contrast, in a survey of 360 physicians in Brazil, 95% of physicians reported prescribing LDA for preeclampsia prevention (26). A number of studies examined the role of a higher dose of aspirin in prevention of preeclampsia; one study in India randomized patients to 150 mg daily of aspirin vs. 75 mg daily and found significantly lower rates of pre-eclampsia in the higher dose group (27). The dose effect of LDA is still debated and being investigated. To date, there is not enough evidence to recommend the use of higher LDA doses.

#### 3.1.3 Vitamins and supplements

A total of 10 studies addressed the impacts of various vitamins and supplements, including vitamin D, vitamin C, folic acid, magnesium, selenium, iron, and arginine. Five studies revealed no significant impact of vitamins and supplements on rates of HDP, supporting the 2008 Cochrane review demonstrating that antioxidant supplementation does not reduce the risk of pre-eclampsia (28). One study in China revealed that individuals who started the multiple micronutrient supplementation at <12 weeks gestation had a 26% reduction in pregnancy-induced hypertension compared with those who started at ≥12 weeks (29). Two randomized controlled trials in Pakistan (including 90 women) and the Democratic Republic of Congo (including 1,159 women) demonstrated lower rates of preeclampsia in women who received vitamin D supplementation during pregnancy (30, 31).

### 3.2 Screening

A total of 41 studies focused on screening for HDP. Four studies reviewed current screening and treatment practices for HDP. These reviews compared current clinic practices to established international guidelines and found deficiencies in guideline adherence. One study evaluated adherence to WHO standards for screening and treatment of preeclampsia and eclampsia in Ethiopia, Kenya, Madagascar, Mozambique, Rwanda, and Tanzania. Overall, BP measurement of antenatal care clients ranged from 46%–96% and screening for signs of preeclampsia and eclampsia was 11%–34% (42).

A total of 14 studies focused on improving screening for HDP evaluated community-level interventions, including task-sharing with community health workers (CHW), home blood pressure (BP) monitoring, and education programs in the community (Table 1). The primary outcome of these interventions varied from feasibility and acceptability to maternal and perinatal morbidity and mortality. The studies focused on task-sharing with CHW included CHW measuring BP in the community as well as teaching home BP monitoring to patients. CHW were able to assess blood pressure in the community and identify dangerous BP levels and symptoms of HDP (32). Community members found CHW screening for HDP highly acceptable. Studies also found CHW had low baseline knowledge of preeclampsia and eclampsia, suggesting that robust training is essential (33). One study demonstrated support for self-monitoring of BPs at home (34). In a study assessing home BP monitoring in Nigeria, 81%–88% of participants reported that a BP monitoring program would be acceptable, while 47%–69% reported that it would be feasible. Feasibility barriers included inadequate patient and provider knowledge, lack of sustainable financial support, and limited transportation networks for follow-up. Facilitators included simplicity, trusting patient-provider relationships, and availability of mobile technology for linkage to care (35). The community-level interventions for preeclampsia (CLIP) intervention package, consisting of CHW-led home visits for detection and management of HDP as well as community engagement sessions to improve knowledge and awareness of HDP, was tested in a number of countries, including Mozambique, India, and Pakistan (35–38). One study assessing CLIP in Mozambique found that while the CLIP intervention increased the frequency of routine antenatal care, it did not improve compliance with BP or proteinuria measurement or improve maternal and fetal morbidity and mortality (35).

Five studies focused on trainings for healthcare providers, including nurses, midwives, and doctors, on improving screening for preeclampsia and eclampsia using BP and proteinuria assessment. A study in Bolivia compared rates of screening for and diagnosis of HDP before and after a workshop for providers covering The American College of Obstetricians and Gynecologists (ACOG) diagnostic guidelines for HDP; the workshop improved adherence with ACOG diagnostic guidelines (39). A study from India found that a simulation-based training for nurses on preeclampsia and eclampsia diagnosis and management increased screening for HDP clinical symptoms and adherence to management steps (40). Finally, a study from Zanzibar implemented a training seminar and pocket reference guide to improve diagnosis and management of HDP; this training did not increase BP monitoring of laboring women, but it improved diagnosis of severe HDP (severe hypertension and/or eclampsia) from 6.8% to 8.2% and increased BP monitoring in the postpartum period from 67% to 77% (41).

Finally, five studies focused on mobile health interventions to improve screening for and diagnosis of HDP. Interventions were found to be feasible and effective in improving patient knowledge about HDP; one study in Iran found a mobile educational application improved women's knowledge about preeclampsia (43).

### 3.3 Treatment

Of the 139 included studies on treatment of HDP, the majority address pharmacologic interventions with 44 investigating magnesium, 39 comparing regimens of anti-hypertensive medications, and 7 on other pharmacologic treatments. Of the remaining studies, 7 compared expectant management (consisting of regular monitoring, antihypertensives, and corticosteroids) vs. planned delivery and 8 focused on alternative therapies such as music therapy, relaxation therapy, and acupuncture (Table 1).

#### 3.3.1 Antihypertensives

The results of studies comparing various types of antihypertensives demonstrated mixed results. The most studied medications were nifedipine, hydralazine, methyldopa, and labetalol. Many studies found that short-acting oral nifedipine was either superior or noninferior to intravenous (IV) antihypertensives in achieving BP targets, preventing severe hypertension, and preventing adverse maternal and neonatal outcomes (28). One study in Malaysia compared maternal and neonatal outcomes in patients with sustained severe hypertension treated with IV labetalol vs. oral nifedipine; outcomes demonstrated that nifedipine achieved target BP faster (median 30 min, compared with median 45 min for labetalol), although this difference was not statistically significant, and there were no differences in the number of doses required or pregnancy outcomes (44). Another study, conducted in Nigeria, compared oral nifedipine with IV hydralazine in women with severe hypertension; this study showed faster BP control with hydralazine (mean 44 min) compared with nifedipine (mean 51 min), which was not statistically significant, but patients in the nifedipine group required fewer doses and had a lower risk of recurrence and retreatment. There were no differences in neonatal outcomes between groups (79).

Additionally, a number of studies investigated the role of antihypertensives in patients with mild to moderate hypertension in LMICs, with one study conducted in Egypt showing that treatment with methyldopa or nifedipine reduced the risk of severe hypertension, preeclampsia, renal impairment, ECG changes, placental abruption, and repeated hospital admissions compared to no medication (45).

#### 3.3.2 Magnesium

The efficacy of magnesium sulfate for eclampsia prophylaxis and for the treatment of eclampsia has been well established in its ability to prevent or control seizures; despite this, magnesium sulfate remains underutilized in many LMICs (46–48). A study in India found that the proportion of patients with eclampsia who received magnesium sulfate varied widely across states, from 12%–76%, with lower rates in rural communities. Barriers to administration include lack of appropriate equipment, limited staff awareness, fear of adverse effects, inadequate supply chain, and lack of clinical protocols (49–51). A commonly cited facilitator to increase magnesium sulfate adherence was periodic trainings for healthcare providers. One such study in Nigeria demonstrated a significant decline in the case fatality rate of eclampsia from 15.1% to 3.2% following a training (52).

Other studies have examined shorter dosing regimens of magnesium, such as a single loading dose or 6- and 12-hour regimens compared to the traditional 24-hour regimen (53–65). Most studies demonstrated similar clinical efficacy between the shorter regimen and traditional regimen, including similar seizure rates, maximum BPs, and intensive care unit admissions. Shorter courses of magnesium sulfate may be associated with reductions in adverse effects and length of hospital admission. A study in Ghana randomized 1,176 patients to receive a 12-hour shortened regimen or a 24-hour standard regimen and found no significant differences in seizure rates, maximum BP, or adverse maternal outcomes between groups (55).

#### 3.3.3 Expectant management

Expectant management in patients with preeclampsia with severe features requires close maternal and fetal monitoring with ability to pursue delivery promptly should concerning symptoms arise. Consistent with ACOG guidelines, most of the studies examining expectant management in patients with preeclampsia with severe features demonstrated a benefit of expectant management up to 34 weeks with improved neonatal outcomes compared with immediate delivery (46). One study conducted in India demonstrated that expectant management and non-invasive monitoring to later gestational ages for women with severe preeclampsia correlated with reduction of respiratory distress syndrome and improved neonatal outcomes. It also found no cases of maternal death amongst women managed expectantly and that other maternal complications, such as pulmonary edema, HELLP syndrome, and retinal/cortical edema, resolved within one week (66). After 34 weeks gestation, several studies demonstrated that immediate delivery was associated with a lower risk of severe hypertension and stillbirth compared with expectant management (67, 68). In summary, these studies support expectant management in patients with severe preeclampsia up to 34 weeks; however, after 34 weeks, planned delivery is beneficial in these patients.

#### 3.3.4 Alternative therapies

Finally, the studies on the role of alternative therapies included a number of modalities, primarily music therapy and relaxation therapy, but also acupuncture and Ayurveda (a holistic system of medicine originating in India). Despite small sample sizes, the studies examining the role of music and various forms of relaxation therapy (Benson's, Jacobson's) showed efficacy in reducing BPs amongst women with HDP, including both severe and non-severe preeclampsia and chronic hypertension. A study conducted in India compared Benson's relaxation therapy, a technique which requires women to breathe quietly with their eyes closed for a period of 15–20 min daily, in addition to antihypertensive medications vs. antihypertensives alone and found greater reductions in systolic (mean difference: control 5.9 +/- 5.5, intervention 14.6 +/- 3.8, *p* = 0.000) and diastolic (mean difference: control 5.8 +/- 7.3, intervention 14.0 +/- 3.7, *p* = 0.000) BPs amongst the relaxation therapy group (69)

## 4 Discussion

With each pregnancy comes a risk of HDP and its complications – stroke, end-organ dysfunction, and maternal and fetal death – particularly in low- and middle-income settings, where fertility rates tend to be higher on average than in high-income countries and access to perinatal health care lower (70). Optimal care for pregnant women with HDP necessitates action at every stage of the care continuum, from prevention and screening to diagnosis and treatment, and at each pregnancy during the women's lifecourse. The conceptual framework presented in this review emphasizes these key steps of care, as well as the interconnectedness between steps. Most interventions in this review focused on improving treatment for HDP, with an emphasis on tailoring treatment regimens to local resources, with few addressing challenges of implementing, scaling, and sustaining these interventions. Additionally, studies were lacking in countries bearing the highest burden of disease, such as South Sudan, Chad, Central African Republic, and Haiti.

This review presents a conceptual framework involving distinct stages of prevention, screening, and treatment but also emphasizing that these stages are interconnected and dynamic with women moving easily from prevention to screening to treatment across their pregnancies during their lives. Only 10% of interventions addressed multiple stages of the care continuum. One such intervention is CLIP intervention package, a community-based package of care aimed at improving maternal and neonatal outcomes for preeclampsia. This intervention implemented a CHW program to improve screening for and diagnosis of preeclampsia as well as reduce delays in transport to and treatment at health facilities (35–38). The CLIP intervention effectively improved antenatal care attendance, however, improved access to antenatal care did not always result in improved maternal outcomes (35). Future interventions for HDP should prioritize integrated models of care that encompass the entire care continuum to optimize health outcomes, as all steps are essential for reduced maternal morbidity and mortality.

The vast majority of studies included clinical interventions focused on medications for treatment of HDP. Practical adaptations of medications were assessed using rigorous clinical trial designs, such as the use of oral antihypertensives over intravenous dosing regimens or more convenient dosing algorithms for magnesium sulfate that allow for shorter hospital stays and reduced staffing. Despite these evidence-based pharmacologic interventions, implementation and uptake in LMICs remains poor (71, 72). Medications such as low-dose aspirin, calcium supplements, and antihypertensives are included on the WHO's 2023 model list of essential medicines, but patients continue to face barriers in accessing these treatments, including resource limitations like insufficient staffing and equipment as well as gaps in knowledge for both patients and providers. There was notably an absence of research targeting implementation strategies adapting these evidence-based interventions. Potential implementation strategies, leveraging success in the treatment of HIV in these settings, include decentralization of services into community settings, home BP monitoring, task sharing screening and treatment, the use of technology and mobile health interventions, and strengthening the supply chain may be applicable to increasing availability of medications for HDP (73–75).

**Figure 3:**
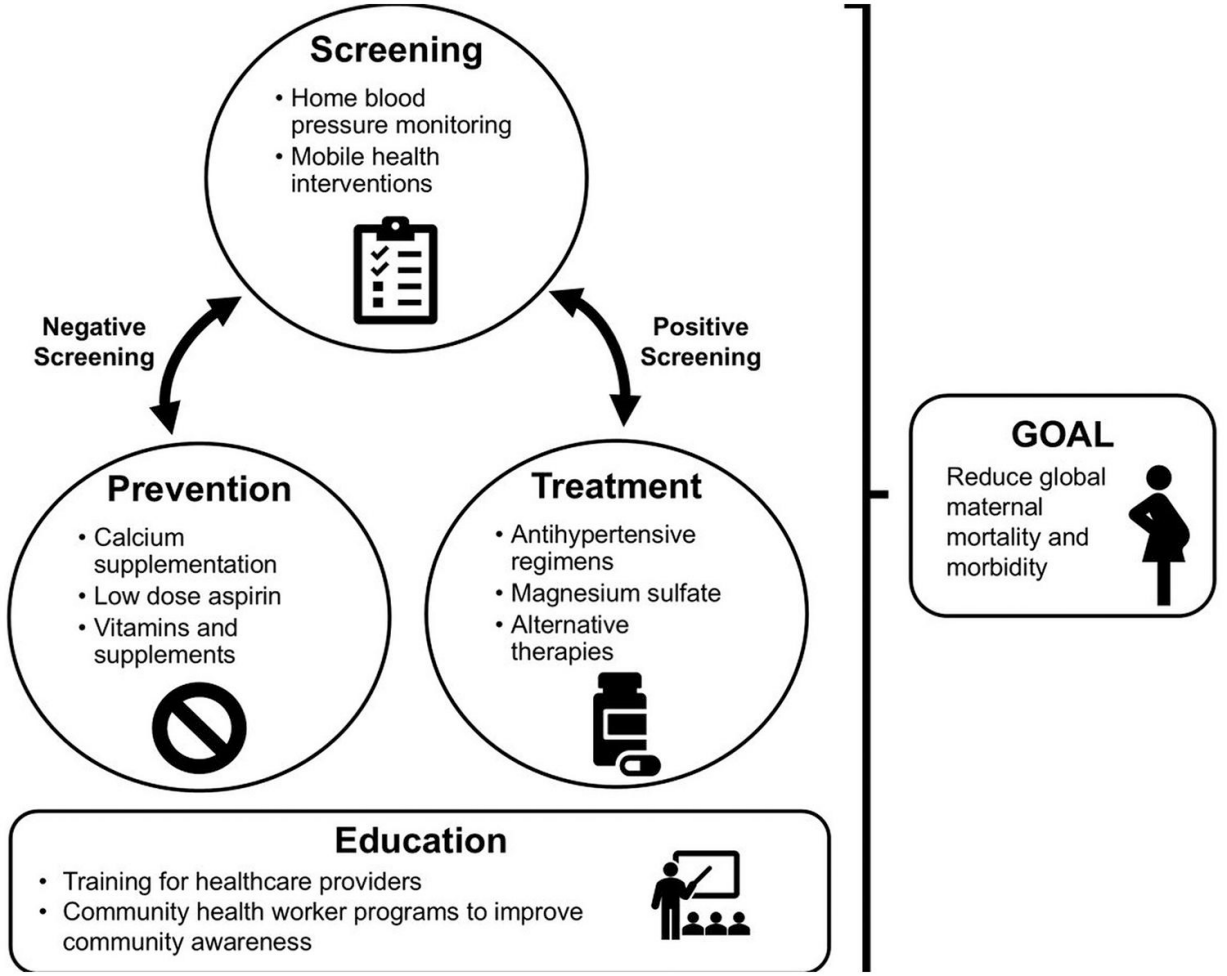
Stages of the care continuum.

Our findings build upon prior reviews on HDP in LMICs that identify persistent limitations to managing these conditions, including limited access to medications and diagnostics, shortages of healthcare providers, and community perceptions and gaps in knowledge related to HDP (8, 76). Our review reveals additional improvements in the availability of prevention measures for HDP, growing evidence for the use of antihypertensives during pregnancy, and expansion of accessibility and use of magnesium sulfate. Prior reviews focused on only low-income countries, while this review included both low- and middle-income countries. Additionally, our review covers recent research on alternative therapies for HDP, including vitamins and supplements for prevention as well as relaxation therapy for treatment. Finally, our review included studies from 2010 to 2024 and presents a conceptual framework to categorize interventions for HDP across the care continuum (Figure 3).

A limitation of this review is lack of available studies from low-income settings, as the majority of studies were from middle-income countries. More countries with the highest rates of HDP (or maternal mortality) such as South Sudan, Chad, Central African Republic, and Haiti are not represented (Figure 2). Identifying this gap is a critical step in encouraging the targeted development of interventions to improve care for HDP in countries with particularly high rates of maternal mortality. Only studies published in English were included in this review, which introduces a potential source of bias. Future multilingual studies are necessary, which would expand knowledge of regional interventions and present an opportunity to adapt effective interventions across countries. Additionally, while the broad scope of this review allows for a comprehensive overview of interventions for HDP, heterogeneity across studies in terms of study design, interventions, and outcomes prevents quantitative analysis of results or formal evaluation of bias. Future studies focusing on a subset of interventions with standardized outcomes are necessary to enable quantitative synthesis and more focused quality evaluation.

In summary, interventions aimed at prevention, screening, and treatment of HDP in LMICs include tailoring medication regimens to local resources, shifting care into the community, and implementing education interventions for both patients and providers. To maximize the impact of these interventions, further research is needed to adapt these evidence-based interventions into implementation strategies for scale and sustainability in order to have population-level improvements in maternal health. Additionally, to comprehensively improve care for HDP, interventions should target all stages of the care continuum, from prevention and screening to diagnosis and treatment. Finally, future research must focus on scaling and adapting interventions across countries to improve care for HDP in settings with the highest rates of maternal mortality globally.

## Data Availability

The original contributions presented in the study are included in the article/Supplementary Material, further inquiries can be directed to the corresponding author.
