# Supplementary material for: Hypertensive disorders of pregnancy in low- and middle-income countries: a review of antenatal interventions to improve maternal outcomes
Source: Front Glob Womens Health. 2026 Jun 24;7:1848249. doi: 10.3389/fgwh.2026.1848249 (PMC13341807; doi:10.3389/fgwh.2026.1848249)
Supplement: Supplementary file 1 [file Table1.docx]

Supplementary Material

# Supplemental File 1. Sample Search Strategy

**Embase (Ovid)**

**Used Cochrane LMIC filter**

(*hypertension/ OR *preeclampsia/ OR *eclampsia/ OR hypertensi*.tw. OR preeclampsia.tw. OR pre-eclampsia.tw. OR eclampsia.tw.) AND (*magnesium/ OR *acetylsalicylic acid/ OR *calcium/ OR magnesium.tw. OR aspirin.tw. OR antiplatelet*.tw. OR anti-platelet*.tw. OR calcium.tw. OR intervention*.tw. OR treatment*.tw. OR management.tw. OR preventative.tw. OR prevention.tw. OR antihypertensive*.tw. OR anti-hypertensive*.tw. OR therap*.tw. OR diagnos*.tw.) AND (*pregnancy/ OR *perinatal period/ OR *prenatal period/ OR pregnan*.tw. OR postpartum.tw. OR antepartum.tw. OR peripartum.tw. OR prenatal.tw. OR prepartum.tw. OR gestation*.tw.) AND (afghan* OR africa* OR albania* OR algeria* OR angola* OR antigua* OR barbuda* OR argentin* OR armenia* OR aruba* OR azerbaijan* OR bahrain* OR bangladesh* OR bengal* OR bangal* OR barbados* OR barbadian* OR bajan OR bajans OR belarus* OR belorus* OR byelarus* OR byelorus* OR belize* OR benin* OR dahomey OR bhutan* OR bolivia* OR bosnia* OR herzegovin* OR botswan* OR batswan* OR bechuanaland* OR brazil* OR brasil* OR bulgaria* OR burkina* OR burkinese* OR upper volta* OR burundi* OR urundi* OR cabo verde* OR cape verde* OR cambodia* OR kampuchea* OR khmer* OR cameroon* OR cameroun* OR ubangi shari* OR chad* OR chile* OR china* OR chinese OR colombia* OR comoro* OR comore* OR comorian* OR mayotte* OR congo* OR zaire* OR costa rica* OR "cote d'ivoir*" OR "cote d' ivoir*" OR cote divoir* OR cote d ivoir* OR ivory coast* OR ivorian* OR croatia* OR cuba OR cuban OR cubans OR "cuba's" OR cyprus* OR cypriot* OR czech* OR djibouti* OR french somaliland* OR dominica* OR ecuador* OR egypt* OR united arab republic* OR el salvador* OR salvadoran* OR guinea* OR equatoguinea* OR eritrea* OR estonia* OR eswatini* OR swaziland* OR swazi* OR swati* OR ethiopia* OR fiji* OR gabon* OR gabonese* OR gabonaise* OR gambia* OR ((georgia OR georgian OR georgians) NOT (atlanta OR california OR florida)) OR ghana* OR gibraltar* OR greece* OR greek* OR grecian* OR grenada* OR grenadian* OR guam* OR guatemala* OR guyana* OR guiana* OR guyanese* OR haiti* OR hispaniola* OR hondura* OR hungary* OR hungarian* OR india* OR indonesia* OR iran* OR iraq* OR isle of man* OR jamaica* OR jordan* OR kazakh* OR kenya* OR karabati* OR korea* OR kosovo* OR kosova* OR kyrgyz* OR kirgiz* OR kirghiz* OR laos OR lao OR laotian* OR latvia* OR lebanon* OR lebanese* OR lesotho* OR lesothan* OR lesothonian* OR basutoland* OR mosotho* OR basotho* OR liberia* OR libya* OR jamahiriya* OR lithuania* OR macedonia* OR madagasca* OR malagasy* OR malawi* OR nyasaland* OR malaysia* OR malay* federation OR maldives* OR maldivian* OR indian ocean OR mali OR malian* OR "mali's" OR malta OR maltese* OR "malta's" OR micronesia* OR marshallese* OR kiribati* OR marshall island* OR nauru OR nauran OR nauruans OR "naurian's" OR mariana OR marianas OR palau OR paluan* OR tuvalu* OR mauritania* OR mauritan* OR mauritius* OR mexico* OR mexican* OR moldova* OR moldovia* OR mongol* OR montenegr* OR morocco* OR moroccan* OR ifni OR mozambique* OR mozambican* OR myanmar* OR burma* OR burmese OR namibia* OR nepal* OR new caledonia* OR netherlands antill* OR nicaragua* OR niger* OR oman OR omani OR omanis OR "oman's" OR pakistan* OR palestin* OR gaza* OR west bank* OR panama* OR paraguay* OR peru OR peruvian* OR "peru's" OR philippine* OR philipine* OR phillipine* OR phillippine* OR filipino* OR filipina* OR poland* OR polish OR pole OR poles OR portugal* OR portuguese OR puerto ric* OR romania* OR russia* OR ussr* OR soviet* OR rwanda* OR rwandese OR ruanda* OR ruandese OR samoa* OR navigator island* OR pacific island* OR polynesia* OR "sao tome and principe*" OR sao tomean* OR santomean* OR saudi arabia* OR saudi OR saudis OR senegal* OR serbia* OR seychell* OR sierra leone* OR slovak* OR sloven* OR melanesia* OR solomon island* OR norfolk island* OR somali* OR sri lanka* OR ceylon* OR "saint kitts and nevis*" OR "st kitts and nevis*" OR kittian* OR nevisian* OR saint lucia* OR st lucia* OR saint vincent* OR st vincent* OR vincentian* OR grenadine* OR sudan* OR surinam* OR syria* OR tajik* OR tadjik* OR tadzhik* OR tanzania* OR tanganyika* OR thai* OR timor leste* OR east timor* OR timorese* OR togo OR togoles* OR "togo's" OR tonga* OR trinidad* OR tobago* OR tunisia* OR turkiy* OR turkey* OR turk OR turks OR turkish OR turkmen* OR uganda* OR ukrain* OR uruguay* OR uzbek* OR vanuatu* OR new hebrides* OR venezuela* OR vietnam* OR viet nam* OR yemen* OR yugoslav* OR zambia* OR zimbabwe* OR rhodesia* OR arab* countr* OR middle east* OR global south OR sahara* OR subsahara* OR magreb* OR maghrib* OR west indies* OR caribbean* OR central america* OR latin america* OR south america* OR central asia* OR north asia* OR northern asia* OR southeastern asia* OR south eastern asia* OR southeast asia* OR south east asia* OR west asia* OR western asia* OR east europe* OR eastern europe* OR developing countr* OR developing nation* OR developing population* OR developing world OR less developed countr* OR less developed nation* OR less developed world OR lesser developed countr* OR lesser developed nation* OR lesser developed world OR under developed countr* OR under developed nation* OR under developed world OR underdeveloped countr* OR underdeveloped nation* OR underdeveloped world OR middle income countr* OR middle income nation* OR middle income population* OR low income countr* OR low income nation* OR low income population* OR lower income countr* OR lower income nation* OR lower income population* OR underserved countr* OR underserved nation* OR underserved population* OR under served population* OR under served nation* OR under served population* OR deprived countr* OR deprived population* OR high burden countr* OR high burden nation* OR countdown countr* OR countdown nation* OR poor countr* OR poor nation* OR poor population* OR poor world OR poorer countr* OR poorer nation* OR poorer population* OR poorer world OR developing econom* OR less developed econom* OR underdeveloped econom* OR under developed econom* OR middle income econom* OR low income econom* OR lower income econom* OR low gdp OR low gnp OR low gross domestic OR low gross national OR lower gdp OR lower gnp OR lower gross domestic OR lower gross national OR lmic OR lmics OR third world OR lami countr* OR transitional countr* OR emerging econom* OR emerging nation*).ti,ab,hw,kf.

# Supplemental File 2. Included Studies

1. KBC R, RG B, DL R, et al. External validation of first trimester combined screening for pre-eclampsia in Brazil: an observational study. Pregnancy Hypertens. 26:110-115. doi:10.1016/j.preghy.2021.10.005

2. Randive JH, McMahon MC, Christmas A, et al. Simulation-enhanced nurse mentoring to improve preeclampsia and eclampsia care: an education intervention study in Bihar, India. BMC Pregnancy Childbirth. 19(1):41. doi:10.1186/s12884-019-2186-x

3. Valiani M, Bahadoran P, Azizi M, Naseh Z. The effect of body relaxation techniques on pre-eclampsia syndrome. Iran J Nurs Midwifery Res. 28(3):320-325. doi:10.4103/ijnmr.IJNMR_250_20

4. Buitendyk M, Kosgei W, Thorne J, et al. Impact of free maternity services on outcomes related to hypertensive disorders of pregnancy at Moi Teaching and Referral Hospital in Kenya: a retrospective analysis. BMC Pregnancy Childbirth. 23(1):98. doi:10.1186/s12884-023-05381-3

5. Jaber OH, Abdulridha WI. Role of calcium supplementation on pregnancy induced hypertension outcomes. Indian J Forensic Med Toxicol. 16(3):280-286. doi:10.37506/ijfmt.v16i3.18298

6. Adebayo JA, Nwachukwu JI, Loto LO, Ezechi CO, Oladapo AA. Efficacy of nifedipine versus hydralazine in the management of severe hypertension in pregnancy: a randomised controlled trial. Niger Postgrad Med J. 27(4):317-324. doi:10.4103/npmj.npmj_275_20

7. Rawlins B, Plotkin M, Rakotovao JP, et al. Screening and management of pre-eclampsia and eclampsia in antenatal and labor and delivery services: findings from cross-sectional observation studies in six sub-Saharan African countries. BMC Pregnancy Childbirth. 18(1):346. doi:10.1186/s12884-018-1972-1

8. Udoma EA, Bassey FA, Bassey OO, Oladokun A. Intravenous magnesium sulfate in the management of severe pre-eclampsia: a randomized study of 12-hour versus 24-hour maintenance dose. Int J Gynaecol Obstet. 149(1):37-42. doi:10.1002/ijgo.13082

9. Muhammad S, Usman H, Dambam YM, Yahya A, Yekeen A, Bako B. Comparison of intravenous labetalol and hydralazine for severe hypertension in pregnancy in Northeastern Nigeria: a randomized controlled trial. Pregnancy Hypertens. 29:1-6. doi:10.1016/j.preghy.2022.05.001

10. Chen Y, Liu Y, Liu M, Wang Y, Zhao H, Huang L. Effects of magnesium sulfate combined with compound danshen injection on pregnancy outcome, vascular endothelia function, liver and kidney function in patients with EOSPE. Int J Clin Exp Med. 13(5):3703-3709.

11. Jiang F, Liu Y, Xu P, et al. The efficacy of the dietary approaches to stop hypertension diet with respect to improving pregnancy outcomes in women with hypertensive disorders. J Hum Nutr Diet. 32(6):713-718. doi:10.1111/jhn.12654

12. Darngawn L, Jose R, Regi A, Bansal R, Jeyaseelan L. A shortened postpartum magnesium sulfate prophylaxis regime in pre-eclamptic women at low risk of eclampsia. Int J Gynaecol Obstet. 116(3):237-239. doi:10.1016/j.ijgo.2011.09.028

13. Gupta A, Nayak D, Sharma J, Keepanasseril A. Comparing the efficacy of oral labetalol with oral amlodipine in achieving blood pressure control in women with postpartum hypertension: randomized controlled trial (HIPPO study). J Hum Hypertens. 37(12):1056-1062. doi:10.1038/s41371-023-00841-x

14. Tukur J, Muhammad Z. Management of eclampsia at AKTH: before and after magnesium sulphate. Niger J Med. 19(1):104-107. doi:10.4314/njm.v19i1.52492

15. Williams A, Koenig MA, Moniruzzaman M, et al. Management of preeclampsia, severe preeclampsia, and eclampsia at primary care facilities in Bangladesh. Glob Health Sci Pract. 7(3):457-468. doi:10.9745/GHSP-D-19-00124

16. Wang Y, Bao J, Peng M. Effect of magnesium sulfate combined with labetalol on serum sFlt-1/PlGF ratio in patients with early-onset severe pre-eclampsia. Exp Ther Med. 20(6):276. doi:10.3892/etm.2020.9406

17. Yin XJ, Yang ZH. Efficacy of nifedipine tablets plus aspirin in patients with gestational hypertension and the effect on coagulation function and hemorheology. Am J Transl Res. 13(6):7059-7064.

18. Sahoo MA, Rout K, Bhusan A. Effectiveness of specific alternative therapy on blood pressure among antenatal mothers with pregnancy-induced hypertension in a selected hospital of Bhubaneswar, Odisha. NeuroQuantology. 20(17):1032-1037. doi:10.48047/NQ.2022.20.17.NQ880132

19. Pan Z, Zhou J, Xu Y. Comparison of outcomes following intravenous magnesium compared with intravenous labetalol and oral nifedipine in 355 pregnant Han Chinese women with preeclampsia. Medicine (United States). 102(46):e35334. doi:10.1097/MD.0000000000035334

20. Shi DD, Yang FZ, Zhou L, Wang N. Oral nifedipine vs. intravenous labetalol for treatment of pregnancy-induced severe pre-eclampsia. J Clin Pharm Ther. 41(6):657-661. doi:10.1111/jcpt.12439

21. Ugwu EO, Dim CC, Obi CD, Nwankwo TO. Maternal and perinatal outcome of severe pre-eclampsia in Enugu, Nigeria after introduction of magnesium sulfate. Niger J Clin Pract. 14(4):418-421. doi:10.4103/1119-3077.91747

22. Sisay AF, Gudu W, Tilahun LB, Birara M, Bekele D. Missed opportunity for aspirin prophylaxis for preeclampsia prevention: a cross-sectional study from Sub-Saharan Africa. AJOG Glob Rep. 4(1):100295. doi:10.1016/j.xagr.2023.100295

23. Nathan HL, Seed PT, Hezelgrave NL, et al. Early warning system hypertension thresholds to predict adverse outcomes in pre-eclampsia: a prospective cohort study. Pregnancy Hypertens. 12:183-188. doi:10.1016/j.preghy.2017.11.003

24. Kabuyanga RK, Tshilombo PL, Sifa B, et al. Effect of early vitamin D supplementation on the incidence of preeclampsia in primigravid women: a randomised clinical trial in Eastern Democratic Republic of the Congo. BMC Pregnancy Childbirth. 24(1):107. doi:10.1186/s12884-024-06277-6

25. Zhao FF, Ai F, Wu J, Dong XD. Changes and clinical significance of serum inflammatory factors in the treatment of pregnancy hypertension syndrome with magnesium sulfate combined with nifedipine. Exp Ther Med. 20(2):1796-1802. doi:10.3892/etm.2020.8863

26. Shi DD, Guo JJ, Zhou L, Wang N. Epigallocatechin gallate enhances treatment efficacy of oral nifedipine against pregnancy-induced severe pre-eclampsia: a double-blind, randomized and placebo-controlled clinical study. J Clin Pharm Ther. 43(1):21-25. doi:10.1111/jcpt.12597

27. Malapaka SV, Ballal PK. Low-dose magnesium sulfate versus Pritchard regimen for the treatment of eclampsia and imminent eclampsia. Int J Gynaecol Obstet. 115(1):70-72. doi:10.1016/j.ijgo.2011.05.013

28. Ramadurg U, Vidler M, Charanthimath U, et al. Community health worker knowledge and management of pre-eclampsia in rural Karnataka State, India. Reprod Health. 13(Suppl 2):113. doi:10.1186/s12978-016-0219-8

29. Liu Y, Li N, Mei Z, et al. Effects of prenatal micronutrients supplementation timing on pregnancy-induced hypertension: secondary analysis of a double-blind randomized controlled trial. Matern Child Nutr. 17(3):e13157. doi:10.1111/mcn.13157

30. Ma L, Li L, Han P, Meng FC, Jiao CH, Zhang HM. Effect of the drug combination of magnesium sulfate and phentolamine on homocysteine and C-reactive protein in the serum of patients with pregnancy-induced hypertension syndrome. Exp Ther Med. 17(5):3682-3688. doi:10.3892/etm.2019.7385

31. Kavi A, Hoffman MK, Swamy MK, et al. Aspirin delays the onset of hypertensive disorders of pregnancy among nulliparous pregnant women: a secondary analysis of the ASPIRIN trial. BJOG. 130(Suppl 3):16-25. doi:10.1111/1471-0528.17607

32. Mahmoud Z, Obi AA, Okafor CF, et al. Facilitators and barriers to optimal home blood pressure management in patients with hypertensive disorders of pregnancy in a tertiary care facility in Abuja, Nigeria: a qualitative research study. BMC Health Serv Res. 23(1):954. doi:10.1186/s12913-023-09976-6

33. Rehman H, Zafar F, Saleem S. Oral labetalol versus oral methyldopa for the management of pregnancy induced hypertension. Pak J Med Health Sci. 15(4):793-795.

34. Biswas SM, Kashem ANS, Rashid SM, et al. Competency of health workers in detecting and managing gestational hypertension, pre-eclampsia, severe pre-eclampsia and eclampsia during antenatal check-ups in primary care health facilities in Bangladesh: a cross-sectional study. BMJ Open. 11(7):e046638. doi:10.1136/bmjopen-2020-046638

35. Kumar A, Seetha K. Prediction by fullPIERS model (Preeclampsia Integrated Estimate of Risk) in preeclampsia patients for adverse maternal and neonatal outcomes. J S Asian Feder Obstet Gynaecol. 15(2):147-152. doi:10.5005/jp-journals-10006-2092

36. Thapa K, Sanghvi H, Rawlins B, et al. Coverage, compliance, acceptability and feasibility of a program to prevent pre-eclampsia and eclampsia through calcium supplementation for pregnant women: an operations research study in one district of Nepal. BMC Pregnancy Childbirth. 16(1):241. doi:10.1186/s12884-016-1033-6

37. Xiang XW, Wang F, Zhao N, Zhou Z. Treatment of pregnancy-induced hypertension compared with labetalol, low dose aspirin and placebo. Cell Mol Biol (Noisy-le-grand). 66(8):9-13. doi:10.14715/cmb/2020.66.8.2

38. Tukur J, Ahonsi B, Ishaku SM, Araoyinbo I, Okereke E, Babatunde AO. Maternal and fetal outcomes after introduction of magnesium sulphate for treatment of preeclampsia and eclampsia in selected secondary facilities: a low-cost intervention. Matern Child Health J. 17(7):1191-1198. doi:10.1007/s10995-012-1105-9

39. Xiao Y, Ling Q, Yao M, et al. Aspirin 75 mg to prevent preeclampsia in high-risk pregnancies: a retrospective real-world study in China. Eur J Med Res. 28(1):56. doi:10.1186/s40001-023-01024-7

40. Macedo SB, Katz L, Nogueira CN, Coutinho BV, Amorim AP, Amorim MM. Abbreviated (12-hour) versus traditional (24-hour) postpartum magnesium sulfate therapy in severe pre-eclampsia. Int J Gynaecol Obstet. 126(3):260-264. doi:10.1016/j.ijgo.2014.03.024

41. El-Khayat W, Atef A, Abdelatty S, El-Semary A. A novel protocol for postpartum magnesium sulphate in severe pre-eclampsia: a randomized controlled pilot trial. J Matern Fetal Neonatal Med. 29(1):154-158. doi:10.3109/14767058.2014.991915

42. el-Sherbiny AH, Fawzy M, El-Negeri M, Nezar M, Ragab A, Halawa AS. Is expectant management of early-onset severe preeclampsia worthwhile in low-resource settings? Arch Gynecol Obstet. 282(1):23-27. doi:10.1007/s00404-009-1209-7

43. Sotunsa OG, Okoro A, Okolo M, et al. Implementing a community-level intervention to control hypertensive disorders in pregnancy using village health workers: lessons learned. Implement Sci Commun. 1:84. doi:10.1186/s43058-020-00076-8

44. von Dadelszen P, Bhutta ZA, Sharma S, et al. The community-level interventions for pre-eclampsia (CLIP) cluster randomised trials in Mozambique, Pakistan, and India: an individual participant-level meta-analysis. Lancet. 396(10250):553-563. doi:10.1016/S0140-6736(20)31128-4

45. Farooq N, Mazhar H, Khan W, Farooq J, Kalsoom S. A comparison of tablet nifedipine with tablet methyldopa in controlling mild to moderate pregnancy induced hypertension (PIH). Pak J Med Health Sci. 14(3):1469-1471.

46. Kebede GM, Negussie D, Alemu JH. Maternal outcomes of magnesium sulphate and diazepam use in women with severe pre-eclampsia and eclampsia in Ethiopia. Pharm Pract (Granada). 12(2):400. doi:10.4321/s1886-36552014000200006

47. Gu W, Lin J, Hou YY, et al. Effects of low-dose aspirin on the prevention of preeclampsia and pregnancy outcomes: a randomized controlled trial from Shanghai, China. Eur J Obstet Gynecol Reprod Biol. 248:156-163. doi:10.1016/j.ejogrb.2020.03.038

48. Hofmeyr GJ, Betrán AP, Singata-Madliki M, et al. Prepregnancy and early pregnancy calcium supplementation among women at high risk of pre-eclampsia: a multicentre, double-blind, randomised, placebo-controlled trial. Lancet. 393(10169):330-339. doi:10.1016/S0140-6736(18)31818-X

49. Zhu H, Yang YJ, Xu Y, et al. Effect of a quality improvement intervention with safety-based checklists for perinatal health of hypertension disorders in pregnancy. Int J Gynaecol Obstet. 157(2):375-382. doi:10.1002/ijgo.13862

50. Chang R, Miao H, Cui A, Jiang L, Yang L, Miao C. Clinical effect of nimodipine combined with magnesium sulfate on pregnancy-induced hypertension syndrome. J Healthc Eng. 2022:7217543. doi:10.1155/2022/7217543

51. Kiondo P, Wamuyu-Maina G, Wandabwa J, Bimenya GS, Tumwesigye NM, Okong P. The effects of vitamin C supplementation on pre-eclampsia in Mulago Hospital, Kampala, Uganda: a randomized placebo controlled clinical trial. BMC Pregnancy Childbirth. 14:283. doi:10.1186/1471-2393-14-283

52. Gomes ZA da S, Marques WR, Souza-Hamann PJ, et al. The WHO Safe Childbirth Checklist implementation: impact on the prescription of magnesium sulphate through a one-year longitudinal study. BMC Pregnancy Childbirth. 20(1):154. doi:10.1186/s12884-020-2836-z

53. Sultana N, Begum K, Begum A. A lower dose of magnesium sulphate for control of convulsion in eclamptic women of Bangladesh. Bangladesh J Obstet Gynaecol. 25(2):71-76. doi:10.3329/bjog.v25i2.13743

54. Alexander D, Northcross A, Wilson N, et al. Randomized controlled ethanol cookstove intervention and blood pressure in pregnant Nigerian women. Am J Respir Crit Care Med. 195(12):1629-1639. doi:10.1164/rccm.201606-1177OC

55. Li Z, Ye R, Zhang L, Li H, Liu J, Ren A. Folic acid supplementation during early pregnancy and the risk of gestational hypertension and preeclampsia. Hypertension. 61(4):873-879. doi:10.1161/HYPERTENSIONAHA.111.00230

56. Atluri N, Beyuo TK, Owusu SA, Danso CD, Moyer CA, Lartey EK. Benefits and barriers of home blood pressure monitoring in pregnancy: perspectives of obstetric doctors from a Ghanaian tertiary hospital. BMC Pregnancy Childbirth. 23(1):42. doi:10.1186/s12884-023-05363-5

57. Toker E. Effect of Turkish classical music on prenatal anxiety and satisfaction: a randomized controlled trial in pregnant women with pre-eclampsia. Complement Ther Med. 30:1-9. doi:10.1016/j.ctim.2016.11.005

58. Cluver CA, Hall NJ, van Papendorp E, et al. Esomeprazole to treat women with preterm preeclampsia: a randomized placebo controlled trial. Am J Obstet Gynecol. 219(4):388.e1-388.e17. doi:10.1016/j.ajog.2018.07.019

59. Nathan HL, Boene H, Munguambe K, et al. The CRADLE vital signs alert: qualitative evaluation of a novel device designed for use in pregnancy by healthcare workers in low-resource settings. Reprod Health. 15(1):5. doi:10.1186/s12978-017-0450-y

60. Chang LH, Liu YF, Zhang XX, et al. The clinical effect of aspirin combined with low-molecular-weight heparin in the treatment of severe preeclampsia and the combination's effect on pregnancy outcomes. Am J Transl Res. 13(8):9113-9121.

61. van Dijk MG, de Oliveira C, Zuspan PU, Galhardo RL, Garite ME, Gloeb SG. Use of magnesium sulfate for treatment of pre-eclampsia and eclampsia in Mexico. Int J Gynaecol Obstet. 121(2):110-114. doi:10.1016/j.ijgo.2012.11.015

62. Karim A, Mannan SK. Role of high dose calcium in the prevention of preeclampsia. Bangladesh J Obstet Gynaecol. 28(2):66-70. doi:10.3329/bjog.v28i2.30092

63. Ogunbode BO, Garba KK, Ibrahim HM. The efficacy of 10 gram intramuscular loading dose of MgSO4 in severe preeclampsia/eclampsia at a tertiary referral centre in Northwest Nigeria. Niger Postgrad Med J. 19(3):143-148.

64. Ghorbannejad S, MehdizadehTourzani Z, Kabir K, MansourehYazdkhasti. The effectiveness of Jacobson's progressive muscle relaxation technique on maternal, fetal and neonatal outcomes in women with non-severe preeclampsia: a randomized clinical trial. Heliyon. 8(6):e09709. doi:10.1016/j.heliyon.2022.e09709

65. Ferreira AS, Seto MTY, Yasmin H, et al. Exploring caregivers' perspectives and perceived acceptability of a mobile-based telemonitoring program to support pregnant women at high-risk for preeclampsia in Karachi, Pakistan: a qualitative descriptive study. Healthcare (Basel). 11(3). doi:10.3390/healthcare11030392

66. Goldberg GR, Jarjou LMA, Cole TJ. Randomized, placebo-controlled, calcium supplementation trial in pregnant Gambian women accustomed to a low calcium intake: effects on maternal blood pressure and infant growth. Am J Clin Nutr. 98(4):972-982. doi:10.3945/ajcn.113.059923

67. Costa MIMP, Ramos KBC, De Moura FG, et al. The prevalence and perinatal repercussions of preeclampsia after the implementation of a prophylaxis protocol with aspirin. Pregnancy Hypertens. 33:17-21. doi:10.1016/j.preghy.2023.06.001

68. Oguntunde O, Charyeva Z, Cannon M, et al. Factors influencing the use of magnesium sulphate in pre-eclampsia/eclampsia management in health facilities in Northern Nigeria: a mixed methods study. BMC Pregnancy Childbirth. 15:130. doi:10.1186/s12884-015-0554-8

69. Sher N, Mirshekar MA, Zafar H, et al. Effect of lipid-based multiple micronutrients supplementation in underweight primigravida pre-eclamptic women on maternal and pregnancy outcomes: randomized clinical trial. Medicina (Kaunas). 58(12). doi:10.3390/medicina58121772

70. Cao SL, Sun J, Wang YX, Zhao YM, Sheng YB, Xu AG. Music therapy improves pregnancy-induced hypertension treatment efficacy. Int J Clin Exp Med. 9(5):8833-8838.

71. Rezaei Z, Sharbaf FR, Pourmojieb M, Youefzadeh-Fard Y, Motevalian M, Khazaeipour Z. Comparison of the efficacy of nifedipine and hydralazine in hypertensive crisis in pregnancy. Acta Med Iran. 49(11):701-706.

72. Cormick G, Ota ZN, Adegoke SA, et al. Gaps between calcium recommendations to prevent pre-eclampsia and current intakes in one hospital in Argentina. BMC Res Notes. 7:920. doi:10.1186/1756-0500-7-920

73. Britto GP, de Pinho PC, Ferreira DR, et al. Effect of the dietary approaches to stop hypertension (DASH) diet on the development of preeclampsia and metabolic outcomes in pregnant women with pre-existing diabetes mellitus: a randomised, controlled, single-blind trial. J Nutr Sci. 12:e73. doi:10.1017/jns.2023.54

74. Beardmore-Gray A, Vousden N, Seed PT, et al. Planned delivery or expectant management for late preterm pre-eclampsia in low-income and middle-income countries (CRADLE-4): a multicentre, open-label, randomised controlled trial. Lancet. 402(10399):386-396. doi:10.1016/S0140-6736(23)00688-8

75. Syafruddin D, Novri DA, Hamidy Y. Effectiveness of nifedipine, labetalol, and hydralazine as emergency antihypertension in severe preeclampsia: a randomized control trial. F1000Research. 11:1287. doi:10.12688/f1000research.125944.2

76. Drost E, van Lonkhuijzen LR, Meguid T, van den Broek N, Zeck W. Implementing safe motherhood: a low-cost intervention to improve the management of eclampsia in a referral hospital in Malawi. BJOG. 117(12):1553-1557. doi:10.1111/j.1471-0528.2010.02691.x

77. Budhwani H, Shivkumar P, Priya CN, et al. Examining the use of magnesium sulfate to treat pregnant women with preeclampsia and eclampsia: results of a program assessment of emergency obstetric care (EmOC) training in India. J Obstet Gynaecol India. 67(5):330-336. doi:10.1007/s13224-017-0964-9

78. Urio UV, Mbofana F, Rocha BM, et al. Diagnostic performance of placental growth factor in women with suspected preeclampsia attending antenatal facilities in Maputo, Mozambique. Hypertension. 69(3):469-474. doi:10.1161/HYPERTENSIONAHA.116.08547

79. van der Merwe JL, Hall DR, Harvey J. Does a patient information sheet lead to better understanding of pre-eclampsia? A randomised controlled trial. Pregnancy Hypertens. 1(3-4):225-230. doi:10.1016/j.preghy.2011.06.001

80. Jyothi J, Nagalakshmi S, Fernandes P. Effectiveness of antenatal care package on knowledge of pregnancy induced hypertension for antenatal mothers in selected hospitals of Mangalore. Nitte Univ J Health Sci. 3(1):8-10.

81. Zhu YC, Zhou Q. Effects of magnesium sulfate and labetalol combined therapy on blood pressure and pregnancy outcomes in early-onset severe preeclampsia. Int J Clin Exp Med. 10(1):1297-1302.

82. Kitiyodom S. Comparison of the level of magnesium during maintenance between 2 gram and 1 gram per hour infusion in overweight mothers with preeclampsia. J Med Assoc Thai. 99(Suppl 7):S133-S137.

83. Ernawati, Gumilar E, Kuntoro, Soeroso J, Dekker G. Expectant management of preterm preeclampsia in Indonesia and the role of steroids. J Matern Fetal Neonatal Med. 29(11):1736-1740. doi:10.3109/14767058.2015.1059815

84. Raheem IA, Saaid R, Omar SZ, Tan PC. Oral nifedipine versus intravenous labetalol for acute blood pressure control in hypertensive emergencies of pregnancy: a randomised trial. BJOG. 119(1):78-85. doi:10.1111/j.1471-0528.2011.03151.x

85. Erida FM, Emilia O, Gunn J, Licqurish S, Lau P. Challenging the status quo: results of an acceptability and feasibility study of hypertensive disorders of pregnancy (HDP) management pathways in Indonesian primary care. BMC Pregnancy Childbirth. 21(1):507. doi:10.1186/s12884-021-03970-8

86. Roy J, Mukherjee JK, Pal A. Magnesium sulphate versus phenytoin in eclampsia — maternal and foetal outcome: a comparative study. Australas Med J. 6(9):483-495. doi:10.4066/AMJ.2013.1753

87. Sevene E, Boene H, Vidler M, et al. Feasibility of task-sharing with community health workers for the identification, emergency management and referral of women with pre-eclampsia, in Mozambique. Reprod Health. 18(1):145. doi:10.1186/s12978-021-01192-x

88. Suksai M, Geater A, Suntharasaj T, Suwanrath C, Charernjiratragul K, Khwankaew N. Low-dose aspirin for prevention of preeclampsia: implementation of the NICE guideline in Thailand. J Obstet Gynaecol Res. 48(9):2345-2352. doi:10.1111/jog.15343

89. Prasad S, Singhal DS, Vanamail P, Sharma A, Arora S, Kaul A. Performance of Fetal Medicine Foundation algorithm for first trimester preeclampsia screening in an indigenous south Asian population. BMC Pregnancy Childbirth. 21(1):805. doi:10.1186/s12884-021-04283-6

90. Souza EV, Torloni MR, Atallah AN, Santos GM, Kulay L Jr, Sass N. Aspirin plus calcium supplementation to prevent superimposed preeclampsia: a randomized trial. Braz J Med Biol Res. 47(5):419-425. doi:10.1590/1414-431x20143629

91. Aghamohammadi A, Zafari M. Calcium supplementation in pregnancy and prevention of hypertensive disorders in elderly women. ScienceAsia. 41(4):259-262. doi:10.2306/scienceasia1513-1874.2015.41.259

92. Hoodbhoy Z, Siddiqui SS, Qureshi R, et al. Role of community engagement in maternal health in rural Pakistan: findings from the CLIP randomized trial. J Glob Health. 11:04045. doi:10.7189/jogh.11.04045

93. Dubey S, Tewary S, Das A, Mishra A, Pawashe K, Tewary S. Comparison of efficacy of oral and intravenous anti-hypertensive drugs in eclampsia: a randomized trial. (2021).

94. Okereke E, Ahonsi B, Tukur J, Ishaku SM, Obika AB. Benefits of using magnesium sulphate (MgSO4) for eclampsia management and maternal mortality reduction: lessons from Kano State in Northern Nigeria. BMC Res Notes. 5:421. doi:10.1186/1756-0500-5-421

95. Okonofua F, Eno-Obong E, Ntoimo LF, et al. Outcomes of a multifaceted intervention to prevent eclampsia and eclampsia-related deaths in Nigerian referral facilities. Int Health. doi:10.1093/inthealth/ihad044

96. Lin L, Huai J, Li B, et al. A randomized controlled trial of low-dose aspirin for the prevention of preeclampsia in women at high risk in China. Am J Obstet Gynecol. 226(2):251.e1-251.e12. doi:10.1016/j.ajog.2021.08.004

97. Kureshy MW, Sheikh S, Bawani S, et al. 'Now you have become doctors': lady health workers' experiences implementing an mHealth application in rural Pakistan. Front Glob Womens Health. 2:645705. doi:10.3389/fgwh.2021.645705

98. Adaeze AB, Kenfack B, Ifeoma MA, et al. Calcium supplementation in pregnancy: an analysis of potential determinants in an under-resourced setting. PLoS One. 18(10):e0292303. doi:10.1371/journal.pone.0292303

99. Chen J, Huai J, Lin L, Li B, Zhu Y, Yang H. Low-dose aspirin in the prevention of pre-eclampsia in China: postpartum hemorrhage in subgroups of women according to their characteristics and potential bleeding risk. Chin Med J (Engl). 136(5):550-555. doi:10.1097/CM9.0000000000002545

100. Zhang XL, Cheng X, Yang TC, Zhao Q. Efficacy of magnesium sulfate combined with nifedipine for pregnancy-induced hypertension syndrome and its relation to glucose and lipid metabolism. Am J Transl Res. 15(9):5940-5948.

101. Bondzi-Simpson JL, van Niekerk SW, Srofenyoh EK, de Graft DE, Klipstein-Grobusch K. Criteria-based audit of quality of care to women with severe pre-eclampsia and eclampsia in a referral hospital in Accra, Ghana. PLoS One. 10(4):e0125749. doi:10.1371/journal.pone.0125749

102. Omotayo MO, Mwanga SL, Stoltzfus RJ, Osero SE, Mwanga E, Dickin KL. With adaptation, the WHO guidelines on calcium supplementation for prevention of pre-eclampsia are adopted by pregnant women. Matern Child Nutr. 14(2):e12521. doi:10.1111/mcn.12521

103. Xiang CP, Zhou XG, Zheng XX. Magnesium sulfate in combination with nifedipine in the treatment of pregnancy-induced hypertension. Pak J Med Sci. 36(2):21-25. doi:10.12669/pjms.36.2.706

104. Liu FM, Yang HL, Li GY, Zou K, Chen YN. Effect of a small dose of aspirin on quantitative test of 24-h urinary protein in patients with hypertension in pregnancy. Exp Ther Med. 13(1):37-40. doi:10.3892/etm.2016.3924

105. Abas MA, Nassar UI, Khan N, Yousaf MD. Low-dose magnesium sulphate in the control of eclamptic fits: a randomized controlled trial. Arch Gynecol Obstet. 287(1):43-46. doi:10.1007/s00404-012-2523-z

106. Katageri G, Charantimath U, Joshi A, et al. Availability and use of magnesium sulphate at health care facilities in two selected districts of North Karnataka, India. Reprod Health. 15(Suppl 1):91. doi:10.1186/s12978-018-0531-6

107. Inta A, Tongsong T, Srisupundit K. Pregnancy outcomes of conservative management in preeclampsia with severe features. J Clin Med. 12(19). doi:10.3390/jcm12196360

108. Agyemang EO, Ribeiro P, Chiwome C, Tettey CA, Acheampong E, Wajeh Y. Integration of suboptimal health status evaluation as a criterion for prediction of preeclampsia is strongly recommended for healthcare management in pregnancy: a prospective cohort study in a Ghanaian population. EPMA J. 10(3):211-226. doi:10.1007/s13167-019-00183-0

109. Barua A, Mundle S, Bracken H, Easterling T, Winikoff B. Facility and personnel factors influencing magnesium sulfate use for eclampsia and pre-eclampsia in 3 Indian hospitals. Int J Gynaecol Obstet. 115(3):231-234. doi:10.1016/j.ijgo.2011.07.016

110. Boene H, Valá A, Kureshy MW, et al. Implementation of the PIERS on the Move mHealth application from the perspective of community health workers and nurses in rural Mozambique. Front Glob Womens Health. 2:659582. doi:10.3389/fgwh.2021.659582

111. Singh R, Kumar J, Jain A, Puri M. Comparison of intravenous anti-hypertensives for preoperative blood pressure control in hypertensive disorders of pregnancy and effect of oral labetalol. Cureus. 14(12). doi:10.7759/cureus.32858

112. Salama M, Rezk M, Gaber W, et al. Methyldopa versus nifedipine or no medication for treatment of chronic hypertension during pregnancy: a multicenter randomized clinical trial. Pregnancy Hypertens. 17:54-58. doi:10.1016/j.preghy.2019.05.009

113. Trapani A, Gonçalves LF, Trapani TF, Vieira S, Pires M, Pires MMS. Perinatal and hemodynamic evaluation of sildenafil citrate for preeclampsia treatment: a randomized controlled trial. Obstet Gynecol. 128(2):253-259. doi:10.1097/AOG.0000000000001518

114. Okonofua FE, Ogu RN, Fabamwo AO, et al. Training health workers for magnesium sulfate use reduces case fatality from eclampsia: results from a multicenter trial. Acta Obstet Gynecol Scand. 92(6):716-720. doi:10.1111/aogs.12135

115. Meena N, Walia R, Sood S, Meena M, Gupta M. Role of labetalol and methyldopa in newly diagnosed mild hypertension in pregnancy: a prospective drug comparative study in a rural tertiary care hospital in India. Int J Pharm Sci Res. 8(1):226-230. doi:10.13040/IJPSR.0975-8232.8(1).226-30

116. Leal FA, Pereira MA, Oliveira MJ, Sass FG, Camano ML, Camano JG. Obstetrician's risk perception on the prescription of magnesium sulfate in severe preeclampsia and eclampsia: a qualitative study in Brazil. PLoS One. 12(3):e0172602. doi:10.1371/journal.pone.0172602

117. Jiang N, Liu Q, Liu L, Yang WW, Zeng Y. The effect of calcium channel blockers on prevention of preeclampsia in pregnant women with chronic hypertension. Clin Exp Obstet Gynecol. 42(1):79-81.

118. George EO, Akintan DO, Dada IOF, et al. Comparison of Zuspan regimen and its 12-hour modification in women with severe pre-eclampsia and eclampsia in two hospitals in Abeokuta. Pregnancy Hypertens. 32:22-27. doi:10.1016/j.preghy.2023.03.001

119. Vigil-De Gracia P, Reyes Tejada O, Calle Minaca A, et al. Expectant management of severe preeclampsia remote from term: the MEXPRE Latin Study, a randomized, multicenter clinical trial. Am J Obstet Gynecol. 209(5):425.e1-425.e8. doi:10.1016/j.ajog.2013.08.016

120. Hoffman MK, Goudar SS, Kodkany BS, et al. Low-dose aspirin for the prevention of preterm delivery in nulliparous women with a singleton pregnancy (ASPIRIN): a randomised, double-blind, placebo-controlled trial. Lancet. 395(10220):285-293. doi:10.1016/S0140-6736(19)32973-3

121. Talungchit P, Liabsuetrakul T, Lindmark G. Multifaceted intervention to implement indicators of quality of care for severe pre-eclampsia/eclampsia. Int J Gynaecol Obstet. 124(2):106-111. doi:10.1016/j.ijgo.2013.08.005

122. Molvi SN, Mir S, Rana VS, Jabeen F, Malik AR. Role of antihypertensive therapy in mild to moderate pregnancy-induced hypertension: a prospective randomized study comparing labetalol with alpha methyldopa. Arch Gynecol Obstet. 285(6):1553-1562. doi:10.1007/s00404-011-2205-2

123. Parsa S, Khajouei R, Balaee MR, Askarpour BS. Improving the knowledge of pregnant women using a pre-eclampsia app: a controlled before and after study. Int J Med Inform. 125:86-90. doi:10.1016/j.ijmedinf.2019.03.001

124. Swamy MK, Patil K, Nageshu S. Maternal and perinatal outcome during expectant management of severe pre-eclampsia between 24 and 34 weeks of gestation. J Obstet Gynaecol India. 62(4):413-418. doi:10.1007/s13224-012-0293-y

125. Skerrett E, Kommwa E, Maynard K, et al. Evaluation of a low-cost, low-power syringe pump to deliver magnesium sulfate intravenously to pre-eclamptic women in a Malawian referral hospital. BMC Pregnancy Childbirth. 17(1):191. doi:10.1186/s12884-017-1382-9

126. Grill JP, Cralcev C, Coelho JS, Marangoni-Junior M, Santos MP, Camano ML. Validation of the fullPIERS model for prediction of adverse outcomes in preeclampsia at a referral center. Pregnancy Hypertens. 23:112-115. doi:10.1016/j.preghy.2020.11.013

127. Dong X, Dong K. Efficacy of normodyne-magnesium sulfate combination treatment on pregnancy-induced hypertension, and its effect on VEGF and Flt-1 levels. Trop J Pharm Res. 20(10):2155-2161. doi:10.4314/tjpr.v20i10.20

128. Sheikh RA, Qureshi RN, Sheikh S, et al. Potential for task-sharing to Lady Health Workers for identification and emergency management of pre-eclampsia at community level in Pakistan. Reprod Health. 13(Suppl 2):107. doi:10.1186/s12978-016-0214-0

129. Bigdeli M, Zafar S, Assad H, Ghaffar A. Health system barriers to access and use of magnesium sulfate for women with severe pre-eclampsia and eclampsia in Pakistan: evidence for policy and practice. PLoS One. 8(3):e59158. doi:10.1371/journal.pone.0059158

130. Gupta MD. Effects of musical therapy and physiotherapy in pregnancy induced hypertension. Indian J Public Health Res Dev. 10(8):339-343. doi:10.5958/0976-5506.2019.01904.1

131. Chantrapitak P, Thinkhamrop P, Lumbiganon J, Srisuwan U, Sriphirom S. Clinical experiences of intravenous hydralazine and labetalol for acute treatment of severe hypertension in pregnant Thai women. J Clin Pharmacol. 60(12):1662-1670. doi:10.1002/jcph.1685

132. Mwende SL, Wawire V, Ombunda H, et al. Integrating calcium supplementation into facility-based antenatal care services in Western Kenya: a qualitative process evaluation to identify implementation barriers and facilitators. Curr Dev Nutr. 2(11):nzy068. doi:10.1093/cdn/nzy068

133. Kidanto HL, Wangwe P, Kilewo CD, Nystrom L, Lindmark G. Improved quality of management of eclampsia patients through criteria based audit at Muhimbili National Hospital, Dar es Salaam, Tanzania. BMC Pregnancy Childbirth. 12:134. doi:10.1186/1471-2393-12-134

134. Sevene E, Sharma S, Munguambe K, et al. Community-level interventions for pre-eclampsia (CLIP) in Mozambique: a cluster randomised controlled trial. Pregnancy Hypertens. 21:96-105. doi:10.1016/j.preghy.2020.05.006

135. Ye Y, Wen L, Liu X, et al. Low-dose aspirin for primary prevention of adverse pregnancy outcomes in twin pregnancies: an observational cohort study based on propensity score matching. BMC Pregnancy Childbirth. 21(1):786. doi:10.1186/s12884-021-04217-2

136. Ponmozhi G, Keepanasseril A, Mathaiyan J, Manikandan K. Nitric oxide in the prevention of pre-eclampsia (NOPE): a double-blind randomized placebo-controlled trial assessing the efficacy of isosorbide mononitrate in the prevention of pre-eclampsia in high-risk women. J Obstet Gynaecol India. 69(Suppl 2):103-110. doi:10.1007/s13224-018-1100-1

137. Toledo-Jaldin L, Lazo-Vega L, Grau L, et al. Increased adherence to ACOG diagnostic guidelines for HDP following a workshop in Bolivia, a LMIC. Pregnancy Hypertens. 34:19-26. doi:10.1016/j.preghy.2023.09.004

138. Ernawati, Aditiawarman, Rahmawati SN. Antihypertensive choices during pregnancy in limited setting. Pharmacognosy J. 15(2):315-318. doi:10.5530/pj.2023.15.46

139. de Pasquale SD, Velarde R, Reyes O, de La Ossa K. Hydralazine vs labetalol for the treatment of severe hypertensive disorders of pregnancy: a randomized, controlled trial. Pregnancy Hypertens. 4(1):19-22. doi:10.1016/j.preghy.2013.08.001

140. Beyuo TK, Lartey EK, Koranteng EK, Owusu SA. A novel 12-hour versus 24-hour magnesium sulfate regimen in the management of eclampsia and preeclampsia in Ghana (MOPEP study): a randomized controlled trial. Int J Gynaecol Obstet. 159(2):495-504. doi:10.1002/ijgo.14181

141. Agida AA, Vidler M, Agida AT, et al. The ability and safety of community-based health workers to safely initiate lifesaving therapies for pre-eclampsia in Ogun State, Nigeria: an analysis of 260 community treatments with MgSO4 and/or methyldopa. Pregnancy Hypertens. 25:179-184. doi:10.1016/j.preghy.2021.05.005

142. Li J, Zhou WY, Zhuang Y, et al. Efficacy of an enhanced recovery nursing plan as a rooming-in practice for women with preeclampsia post-cesarean section. J Clin Hypertens (Greenwich). 26(2):197-206. doi:10.1111/jch.14771

143. Tun TT, Myint T, Lwin S, et al. Promoting antenatal care services for early detection of pre-eclampsia. WHO South East Asia J Public Health. 1(3):290-298. doi:10.4103/2224-3151.207025

144. Zhao Y, Li B, Li T, Chen Y, Chen G. Effects of acupuncture on preeclampsia in Chinese women: a pilot prospective cohort study. Acupunct Med. 34(2):144-148. doi:10.1136/acupmed-2015-010893

145. Kavi A, Kureshy MW, Ranjit UY, et al. Community engagement for birth preparedness and complication readiness in the community level interventions for pre-eclampsia (CLIP) trial in India: a mixed-method evaluation. BMJ Open. 12(12):e060593. doi:10.1136/bmjopen-2021-060593

146. Trishla, Raghvendra A. A comparative study of labetalol vs methyldopa in the treatment of hypertensive disorders of pregnancy. Int J Pharm Clin Res. 14(9):961-967.

147. Charanthimath U, Vidler M, Katageri G, et al. The feasibility of task-sharing the identification, emergency treatment, and referral for women with pre-eclampsia by community health workers in India. Reprod Health. 15(Suppl 1):101. doi:10.1186/s12978-018-0532-5

148. Liu FM, Zhao M, Wang M, Yang HL, Li L. Effect of regular oral intake of aspirin during pregnancy on pregnancy outcome of high-risk pregnancy-induced hypertension syndrome patients. Eur Rev Med Pharmacol Sci. 20(23):5013-5016.

149. Baqai S, Tufail S, Waheed A, Hussain QUA. Optimising preeclampsia first-trimester screening using three parameters. J Coll Physicians Surg Pak. 33(9):995-1000. doi:10.29271/jcpsp.2023.09.995

150. Cozzolino EB, Munhoz LF, Schultz CM, et al. Survey of calcium supplementation to prevent preeclampsia: the gap between evidence and practice in Brazil. BMC Pregnancy Childbirth. 13:206. doi:10.1186/1471-2393-13-206

151. Bagheri FK, Miraj S. The impact of silymarin on improvement of hepatic abnormalities in patients with severe preeclampsia: a randomized clinical trial. Electron Physician. 9(8):5098-5106. doi:10.19082/5098

152. Dwarkanath P, Muhihi A, Sudfeld CR, et al. Two randomized trials of low-dose calcium supplementation in pregnancy. N Engl J Med. 390(2):143-153. doi:10.1056/NEJMoa2307212

153. Rijal MC, Aggrawal A, Pradhan T, Rijal P, Subedi A, Uprety D. Loading dose versus standard regimen of magnesium sulphate in eclampsia: a randomized trial. Nepal Med Coll J. 12(4):244-247.

154. Gerth-Guyette E, Adu-Gyasi D, Terlouw AC, et al. Evaluation of a protein-to-creatinine dipstick diagnostic test for proteinuria screening in selected antenatal care clinics in three districts in the Bono-East Region of Ghana. Pregnancy Hypertens. 30:21-30. doi:10.1016/j.preghy.2022.07.004

155. de Almeida CAL, Ramos JG, Feijo JN, Abreu JG. BRAzil magnesium (BRAMAG) trial: a double-masked randomized clinical trial of oral magnesium supplementation in pregnancy. BMC Pregnancy Childbirth. 20(1):234. doi:10.1186/s12884-020-02935-7

156. Melo BR, Mbofana F, Loquiha O, et al. Early diagnosis of preeclampsia using placental growth factor: an operational pilot study in Maputo, Mozambique. Pregnancy Hypertens. 11:26-31. doi:10.1016/j.preghy.2017.12.005

157. Zhang JG, Li J. Efficacy and safety of combination of magnesium sulfate, phentolamine and nifedipine in treatment of patients with hypertensive disorder complicating pregnancy. Exp Ther Med. 18(5):3341-3346. doi:10.3892/etm.2019.7965

158. Mirza HA, Rasool M, Khalid S, Pervez R, Irshad R. Role of vitamin D for the prevention of pre-eclampsia in pregnant women: a randomized controlled trial. Pak J Med Health Sci. 6(1):1086-1088. doi:10.53350/pjmhs221611086

159. Wang X, Wei W, Qi Y, Dong L. Clinical effects of integrated traditional Chinese and western medicine in treating severe preeclampsia and its influence on maternal and infant outcomes after cesarean section under combined lumbar and epidural anesthesia. Evid Based Complement Alternat Med. 2021:6366914. doi:10.1155/2021/6366914

160. Ferreira AS, Kor DV, Bragagnolo DN, Saleem S, Bhutta Z, Seto E. Understanding the needs of a mobile phone-based telemonitoring program for pregnant women at high risk for pre-eclampsia: interpretive qualitative description study. JMIR Form Res. 6(2):e32428. doi:10.2196/32428

161. Qureshi RN, Sheikh S, Hoodbhoy Z, et al. Community-level interventions for pre-eclampsia (CLIP) in Pakistan: a cluster randomised controlled trial. Pregnancy Hypertens. 22:109-118. doi:10.1016/j.preghy.2020.07.011

162. Sun L. A mushroom diet reduced the risk of pregnancy-induced hypertension and macrosomia: a randomized clinical trial. Food Nutr Res. 64:1-9. doi:10.29219/fnr.v64.4451

163. Khanam F, Hossain B, Mridha SK, et al. The association between daily 500 mg calcium supplementation and lower pregnancy-induced hypertension risk in Bangladesh. BMC Pregnancy Childbirth. 18(1):406. doi:10.1186/s12884-018-2046-0

164. Wang LX, Xing W, Zheng W. Observation of personalized obstetric care clinical intervention on perinatal pregnancy-induced hypertension. Int J Clin Exp Med. 9(11):22345-22350.

165. Taghizadeh F, Mirzaei F, Ostadi K, et al. Selenium supplementation and the incidence of preeclampsia in pregnant Iranian women: a randomized, double-blind, placebo-controlled pilot trial. Taiwan J Obstet Gynecol. 49(2):181-187. doi:10.1016/S1028-4559(10)60038-1

166. Abdi N, Rozrokh A, Alavi A, et al. The effect of aspirin on preeclampsia, intrauterine growth restriction and preterm delivery among healthy pregnancies with a history of preeclampsia. J Chin Med Assoc. 83(9):852-857. doi:10.1097/JCMA.0000000000000400

167. Bellad MB, Goudar SS, Mallapur AA, et al. Community level interventions for pre-eclampsia (CLIP) in India: a cluster randomised controlled trial. Pregnancy Hypertens. 21:166-175. doi:10.1016/j.preghy.2020.05.008

168. Anjum S, Goel PR, Gupta N, Bano I. Maternal outcome with discontinuation of magnesium sulfate immediately postpartum in severe preeclampsia. J S Asian Feder Obstet Gynaecol. 9(2):84-87. doi:10.5005/jp-journals-10006-1464

169. Hong B, Ding X, Jia HM, Zhang JM. Combination treatment of captopril and prazosin to treat patients with gestational hypertension. Exp Ther Med. 16(4):3694-3702. doi:10.3892/etm.2018.6604

170. Brown JA, Rohner DE, Ali AM, Ahmed T, Islam M, Zlotkin SH. Tablets are preferred and more acceptable than powdered prenatal calcium supplements among pregnant women in Dhaka, Bangladesh. J Nutr. 144(7):1106-1112. doi:10.3945/jn.113.188524

171. Huai J, Lin L, Juan J, et al. Preventive effect of aspirin on preeclampsia in high-risk pregnant women with stage 1 hypertension. J Clin Hypertens (Greenwich). 23(5):1060-1067. doi:10.1111/jch.14149

172. Lurie N, Kurtser M. Investigating the preventive effect of vitamins C and E on preeclampsia in nulliparous pregnant women. J Perinat Med. 48(6):625-629. doi:10.1515/jpm-2019-0469

173. Ehikioya E, Oyelaran OE, Benson MAE, et al. Comparing intravenous labetalol and intravenous hydralazine for managing severe gestational hypertension. Cureus. 15(7):e42332. doi:10.7759/cureus.42332

174. Sinha N, Singh S, Agarwal M, et al. A randomized controlled study comparing the efficacy of 75 mg versus 150 mg aspirin for the prevention of preeclampsia in high-risk pregnant women. Cureus. 15(5):e39752. doi:10.7759/cureus.39752

175. Easterling T, Mundle S, Bracken H, et al. Oral antihypertensive regimens (nifedipine retard, labetalol, and methyldopa) for management of severe hypertension in pregnancy: an open-label, randomised controlled trial. Lancet. 394(10203):1011-1021. doi:10.1016/S0140-6736(19)31282-6

176. Kumar N, Das V, Agarwal A, Pandey A, Agrawal S, Singh A. Pilot interventional study comparing fetomaternal outcomes of 150 mg versus 75 mg aspirin starting between 11 and 14 weeks of pregnancy in patients with high risk of preeclampsia: a randomized control trial. J Obstet Gynaecol India. 70(1):23-29. doi:10.1007/s13224-019-01277-5

177. Wen J, Li X. Effect of magnesium sulfate combined with phentolamine and nifedipine for gestational hypertension and serum levels of LIF and apelin. J Coll Physicians Surg Pak. 29(3):231-234. doi:10.29271/jcpsp.2019.03.231

178. Patel NK, Gadhavi M, Gorasia D, Pandya MR. Comparative evaluation of antihypertensive drugs in the management of pregnancy-induced hypertension. (2014).

179. Keepanasseril A, Maurya DK, Manikandan K, Suriya JY, Habeebullah S, Raghavan SS. Prophylactic magnesium sulphate in prevention of eclampsia in women with severe preeclampsia: randomised controlled trial (PIPES trial). J Obstet Gynaecol. 38(3):305-309. doi:10.1080/01443615.2017.1351931

180. Chen H, Tang Y, Liu C, Liu J, Wang K, Zhang X. Adherence to drug therapy for hypertensive disorders of pregnancy: a cross-sectional survey. Arch Public Health. 78:41. doi:10.1186/s13690-020-00423-0

181. da Costa Ferreira EV, Rodrigues TCGF, Santos VC, Vitor ECA, Cezar RC. Prediction and prevention of preeclampsia by physicians in Brazil: an original study. Front Glob Womens Health. 3:983131. doi:10.3389/fgwh.2022.983131

182. Li P, Zhao J, Gao P. Clinical evaluation of pinggan yiqi yangshen recipe combined with labetalol hydrochloride and magnesium sulfate in the treatment of PIH. Evid Based Complement Alternat Med. 2021:3135043. doi:10.1155/2021/3135043

183. Anjum S, Rajaram GP, Bano I. Short-course postpartum (6-h) magnesium sulfate therapy in severe preeclampsia. Arch Gynecol Obstet. 293(5):983-986. doi:10.1007/s00404-015-3903-y

184. Kaur GC, Birhanu Z, Osero SE, et al. Integrating calcium into antenatal iron-folic acid supplementation in Ethiopia: women's experiences, perceptions of acceptability, and strategies to support calcium supplement adherence. Glob Health Sci Pract. 8(3):413-430. doi:10.9745/GHSP-D-20-00008

185. Vermeulen KJC, Pinas RR, Broekhuis A, et al. Why magnesium sulfate 'coverage' only is not enough to reduce eclampsia: lessons learned in a middle-income country. Pregnancy Hypertens. 22:136-143. doi:10.1016/j.preghy.2020.09.006

186. Liu JH, Yang YK, Liu H, Li QD, Zhou WY. Effect of antioxidants on amelioration of high-risk factors inducing hypertensive disorders in pregnancy. Chin Med J (Engl). 123(18):2548-2554.

187. Akbar MIA, Yolanda A, Pohan RE, et al. INOVASIA study: a randomized open controlled trial to evaluate pravastatin to prevent preeclampsia and its effects on sFlt1/PlGF levels. Am J Perinatol. doi:10.1055/a-1673-5603

188. Chukwudi CM, George AD, Bako B, Musa AG, Atterwahmie A. A shortened versus standard matched postpartum magnesium sulphate regimen in the treatment of eclampsia: a randomised controlled trial. Afr J Reprod Health. 17(3):131-136.

189. Rathi R, Sharma G, Sastry CHS, et al. Effect of Ayurvedic interventions on toxemia of pregnancy (preeclampsia) and fetal outcome: a randomized placebo-controlled trial. J Pharm Res Int. 33(53B):136-148. doi:10.9734/JPRI/2021/v33i53B33690

190. Omotayo MO, Dickin KL, Pelletier DL, Mwanga SL, Kung’u JK, Stoltzfus RJ. Feasibility of integrating calcium and iron-folate supplementation to prevent preeclampsia and anemia in pregnancy in primary healthcare facilities in Kenya. Matern Child Nutr. 14(Suppl 1). doi:10.1111/mcn.12437

191. Anjum S, Goel N, Sharma R, Mohsin Z, Garg N. Maternal outcomes after 12 hours and 24 hours of magnesium sulfate therapy for eclampsia. Int J Gynaecol Obstet. 132(1):68-71. doi:10.1016/j.ijgo.2015.06.056

192. Julien KM, Zash R, Mmalane AM, et al. Anti-hypertensive use for non-severe gestational hypertension in Botswana: a case-control study. Int J Gynaecol Obstet. 156(3):481-487. doi:10.1002/ijgo.13809

193. Huang LJ. Clinical efficacy analysis of nifedipine combined with low molecular weight heparin in the treatment of pregnancy hypertension. Indian J Pharm Sci. 83:79-83. doi:10.36468/pharmaceutical-sciences.spl.375

194. Adewuyi DO, Vidler M, Sotunsa JO, et al. Human resource constraints and the prospect of task-sharing among community health workers for the detection of early signs of pre-eclampsia in Ogun State, Nigeria. Reprod Health. 13(Suppl 2):111. doi:10.1186/s12978-016-0216-y

195. Rahmani AH, Karimi M. The favorable effects of garlic intake on metabolic profiles, hs-CRP, biomarkers of oxidative stress and pregnancy outcomes in pregnant women at risk for pre-eclampsia: randomized, double-blind, placebo-controlled trial. J Matern Fetal Neonatal Med. 28(17):2020-2027. doi:10.3109/14767058.2014.977248

196. Zhou Q, Zhao X, Xu J, et al. Low-dose aspirin in the prevention of preeclampsia in twin pregnancies: a real-world study. Front Cardiovasc Med. 9:964541. doi:10.3389/fcvm.2022.964541

197. Vadillo-Ortega F, Perichart-Perera O, Espino S, et al. Effect of supplementation during pregnancy with L-arginine and antioxidant vitamins in medical food on pre-eclampsia in high risk population: randomised controlled trial. BMJ. 342:d2901. doi:10.1136/bmj.d2901

198. Wang YH, Zhang XY, Han YQ, Yan F, Wu R. Efficacy of combined medication of nifedipine and magnesium sulfate on gestational hypertension and the effect on PAPP-A, VEGF, NO, Hcy and vWF. Saudi J Biol Sci. 26(8):2043-2047. doi:10.1016/j.sjbs.2019.08.012

199. Ding J, Kang Y, Fan YQ, Chen Q. Efficacy of resveratrol to supplement oral nifedipine treatment in pregnancy-induced preeclampsia. Endocr Connect. 6(8):595-600. doi:10.1530/EC-17-0130

200. Beardmore-Gray A, Vousden N, Seed SA, et al. Planned early delivery for late preterm pre-eclampsia in a low- and middle-income setting: a feasibility study. Reprod Health. 18(1):110. doi:10.1186/s12978-021-01159-y

201. Ahmadi G, Aghamohammadi ZS, Jaefari Z, Vakilian S, Saffar M, Hantoushzadeh F. The effect of garlic capsule on the prevention of preeclampsia in high-risk Turkmen pregnant women. Jundishapur J Nat Pharm Prod. 15(1):e60484. doi:10.5812/jjnpp.60484

202. Bhuiyan B, Ahmed N, Uddin MK, Alam MA. Fluid and nutritional management can significantly reduce the mortality of patients with eclampsia in resource poor settings. Bangladesh J Obstet Gynaecol. 27(1):18-20. doi:10.3329/bjog.v27i1.29909

203. Zhang FY, Duan BD, Liu YM, Wang CC. Efficacy of aspirin combined with labetalol on gestational hypertension and effect on serum PAPP-A, APN and HMGB1. Am J Transl Res. 13(12):13750-13758.

204. Shashikala N, Menon J, Sreelakshmi EV, et al. Impact of comprehensive antenatal care to reduce the complications during pregnancy like pregnancy-induced hypertension and intrauterine growth retardation. J S Asian Feder Obstet Gynaecol. 10(4):236-244. doi:10.5005/jp-journals-10006-1598

205. Dhongade S, Darade A, Mangal A. Abbreviated (8 hours) versus traditional (24 hours) postpartum MgSO4 prophylaxis in severe preeclampsia: a randomised control trial. J Clin Diagn Res. 15(9):QC01-QC04. doi:10.7860/JCDR/2021/48570.15320

206. Cluver CA, Hiscock R, Hall DG, et al. Use of metformin to prolong gestation in preterm pre-eclampsia: randomised, double blind, placebo controlled trial. BMJ. 374:n2103. doi:10.1136/bmj.n2103

207. Sarvari F, Dolatian F, Ozgoli K. The effect of Orem's self care model on control of preeclampsia in pregnant women: a randomized clinical trial. Res J Pharm Biol Chem Sci. 7(4):1383-1389.

208. Atef S, Dehghan A, Mohammadi SD, Mirzaee F, Bijani M. The effect of relaxation and cognitive counseling intervention on blood pressure and quality of life among pregnant women with chronic hypertension: randomized controlled trial. Neuropsychiatria Neuropsychol. 17(3-4):187-193. doi:10.5114/nan.2022.124705

209. Shekhar S, Sharma C, Thakur S, Verma S. Oral nifedipine or intravenous labetalol for hypertensive emergency in pregnancy: a randomized controlled trial. Obstet Gynecol. 122(5):1057-1063. doi:10.1097/AOG.0b013e3182a9ea68

210. Maaløe N, Aabakke CB, Housseine N, Meguid T, Bygbjerg IC, van Roosmalen J. Effect of locally tailored clinical guidelines on intrapartum management of severe hypertensive disorders at Zanzibar’s tertiary hospital (the PartoMa study). Int J Gynaecol Obstet. 144(1):27-36. doi:10.1002/ijgo.12692

211. Leal FA, Pereira MA, Oliveira MJ, Sass FG, Camano ML, Camano JG. Situational analysis of facilitators and barriers to availability and utilization of magnesium sulfate for eclampsia and severe preeclampsia in the public health system in Brazil. BMC Pregnancy Childbirth. 16(1):254. doi:10.1186/s12884-016-1055-0

212. Omotayo MO, Dickin KL, Pelletier DL, Mwanga EO, Kung’u JK, Stoltzfus RJ. A simplified regimen compared with WHO guidelines decreases antenatal calcium supplement intake for prevention of preeclampsia in a cluster-randomized noninferiority trial in rural Kenya. J Nutr. 147(10):1986-1991. doi:10.3945/jn.117.251926
